# A systematic review of progress toward unlocking the power of epigenetics in breast cancer: latest updates and perspectives

**DOI:** 10.3389/fphar.2025.1628165

**Published:** 2025-09-16

**Authors:** Ghallab Alotaibi

**Affiliations:** Department of Pharmacology, College of Pharmacy, Shaqra University, Shaqra, Saudi Arabia

**Keywords:** breast cancer, epigenetics, biomarkers, personalized medicine, nanotechnology

## Abstract

Breast cancer (BC) is among the most prevalent malignancies globally. It is progressively acknowledged as a diverse type of cancer, exhibiting considerable differences in its genomic and transcriptomic characteristics. Its growing evidence highlights the substantial role of epigenetic modification in pathogenesis, prognosis and treatment. Cancer and epigenetics are closely linked; abnormal epigenetic changes can influence numerous aspects of cancer biology, including unusual transcription patterns, initiation of cancer, its progression, resistance to drugs, and metastasis. Epigenetic drugs (epi-drugs), including DNA methyltransferase (DNMT) and histone deacetylase (HDAC) inhibitors, serve as promising therapeutic agents, particularly in combination with conventional therapies. Additionally, nanotechnology-assisted epi-drug delivery systems are emerging as innovative approaches to enhance treatment efficacy and reduce systemic toxicity. While several epigenetic biomarkers have shown potential in liquid and tissue biopsies, their clinical validation remains a challenge. The integration of epigenetic insights into personalized medicine could revolutionize BC management, offering more targeted and effective treatment strategies. This systematic review aims to evaluate recent advancement in epigenetic research related to BC, focusing on diagnostic and prognostic biomarkers, epigenetic-based therapies and ongoing clinical trials. A comprehensive literature search was carried out in databases like PubMed, Scopus, and Google Scholar up to January 2025, following PRISMA guidelines. Seventy two (72) studies were included, addressing key aspects of DNA methylation, histone modification, and non-coding RNAs as potential biomarkers for early detection and disease progression monitoring.

## 1 Introduction

Breast cancer (BC) is among the most common cancer diagnosed in the U.S among women. Every year, almost 32% of all newly diagnosed cancers in women are breast cancer (Nwosu and Piccolo, 2024), and it is recognized that there is a hereditary factor associated with the development of BC, with an average age of diagnosis being 62 years and an increased risk observed in black women (Nounou et al., 2015). The majority of breast cancers in women originate in ducts, categorized as ductal carcinoma or in lobes categorized as lobular carcinoma. These breast cancers that remain confined to the milk ducts or lobules are classified as non-invasive. In contrast, invasive breast cancer infiltrates surrounding tissues and exhibits distinct molecular characteristics.

Between 2004 and 2017, the incidence of BC surged across various race and ethnic groups in the US, with the most significant average annual percentage increases noted among non-Hispanic Black women (0.9%), particularly those residing in rural areas (1.2%), lower poverty areas (0.8%), and all regions except the West (0.8%–1.0%). Non-Hispanic Blacks experienced sharper increases for local-stage disease and for certain subgroups with distant-stage disease. Among most subgroups, non-Hispanic Blacks experienced the least reduction in regional-stage disease. Likewise, Hispanic women saw the highest increases in certain subgroups, such as those from areas of greater poverty (0.6%–1.2%) and in the West (0.8%), for both local- and distant-stage disease (Kaur et al., 2022).

Several genetic factors that play a role in cancer progression include high-penetrance genes including BRCA1, BRCA2, p53, PTEN, ATM, NBS1, and LKB1, low penetrance cytochrome genes include CYP1A1, CYP2D6, and CYP19, along with genes from the glutathione S-transferase family such as GSTM1 and GSTP1. Additionally, there are genes related to alcohol and one-carbon metabolism like ADH1C and MTHFR, as well as genes that play a role in DNA repair, including XRCC1, XRCC3, and ERCC4/XPF. Furthermore, there are genes that encode cell signalling molecules such as PR, ER, TNF-α, and heat shock protein 70 (HSP70) (Mahendran et al., 2024). Moreover, additional prevalent risk factors for breast cancer encompass lifestyle habits, hormonal influences, socioeconomic status, age, dietary habits, obesity, and exposure to radiation (Nounou et al., 2015).

Breast cancer (BC) is progressively acknowledged as a diverse type of cancer, exhibiting considerable differences in its genomic and transcriptomic characteristics (Dai et al., 2015). For a long time, the causes of cancer were primarily thought to be genetic anomalies. However, as research has shown that the genome is influenced by epigenetic processes, it has become clear that cancer and epigenetics are closely linked. Abnormal epigenetic changes can impact numerous aspects of cancer biology, including unusual transcription patterns, the initiation of cancer, its progression, resistance to drugs, and metastasis. In contrast to genetic mutations, whose correction are challenging, altered epigenetic processes present more viable options for therapy due to their dynamic and reversible qualities. The field of epigenetics holds significant promise for developing cancer treatments and strategies that can restore the normal function of affected genes (Baylin and Jones, 2016).

Epigenetics refers to a hereditary molecular process influenced by exterior elements that governs gene expression without changing the underlying DNA sequence (Holliday, 1994). BC development is characterized by accumulation of irregular alterations at both genetic and epigenetic levels, ultimately resulting in tumor formation. As a result, epigenetic modifications induced by DNA methylation, histone alterations, nucleosome restructuring, and RNA-mediated gene regulation are recognized for their role in influencing various molecular, cellular, and biological processes related to breast cancer development (Dawson and Kouzarides, 2012). Recent research highlights the involvement of epigenetic disruptions in the key characteristics of breast cancer, such as drug resistance and features associated with stemness (Pasculli et al., 2018).

Methylation of DNA is a recognized epigenetic change entailing covalent attachment of methyl group to cytosine base of CpG dinucleotides, leading to the suppression of transcription (Sulewska et al., 2023). Altered methylation patterns of genes and regulatory proteins have increasingly been recognized as factors in the development of human cancers, including BC (Sulewska et al., 2023). Consequently, assays that analyze methylation are being utilized in research focused on creating new diagnostic and treatment approaches for BC, as demonstrated by multiple studies (Salas et al., 2020).

Histone modifications, including phosphorylation, acetylation, ubiquitination, and methylation can influence the expression of gene by changing chromatin accessibility and the process of gene transcription (Sulewska et al., 2023). Research indicates that modifications of histone acetylation (HAMs) are crucial in the development of BC. Recent investigations into abnormal HAMs have sought to uncover the fundamental molecular mechanisms that contribute to the progression of BC and its treatment outcomes (Guo et al., 2018).

Recently, non-coding RNAs (ncRNAs) have been found to play a role in various epigenetic mechanisms that regulate gene expression, including transcription regulation, post-transcriptional modifications, and the alteration of chromatin structure (Penna et al., 2016). Ongoing research is concentrating on the function of ncRNA in BC (Sher et al., 2022).

Epi-regulation encompasses interactions that are more intricate than standalone occurrences, like the relationship between DNA methylation and miRNAs in silencing protein-coding genes (Yamashita et al., 2015). Interestingly, it has been discovered that over 14% of all miRNA species are regulated by DNA methylation, and the methylation of histone tails has been suggested as another mechanism that affects miRNA genes (Sulewska et al., 2023). Furthermore, miRNAs have the ability to reduce the function of long non-coding RNA (lncRNA), and lncRNA can also be inhibited through the deacetylation of their associated histones (Sun et al., 2014). Additionally, a collection of epi-miRNAs can indirectly affect epigenetic regulators, whereas epigenetic modulators can interact directly with genetic alterations. For instance, DNA methylation accounts for more than 30% of germline point mutations associated with diseases (Liu et al., 2017; Langevin et al., 2015).

**FIGURE 1:**
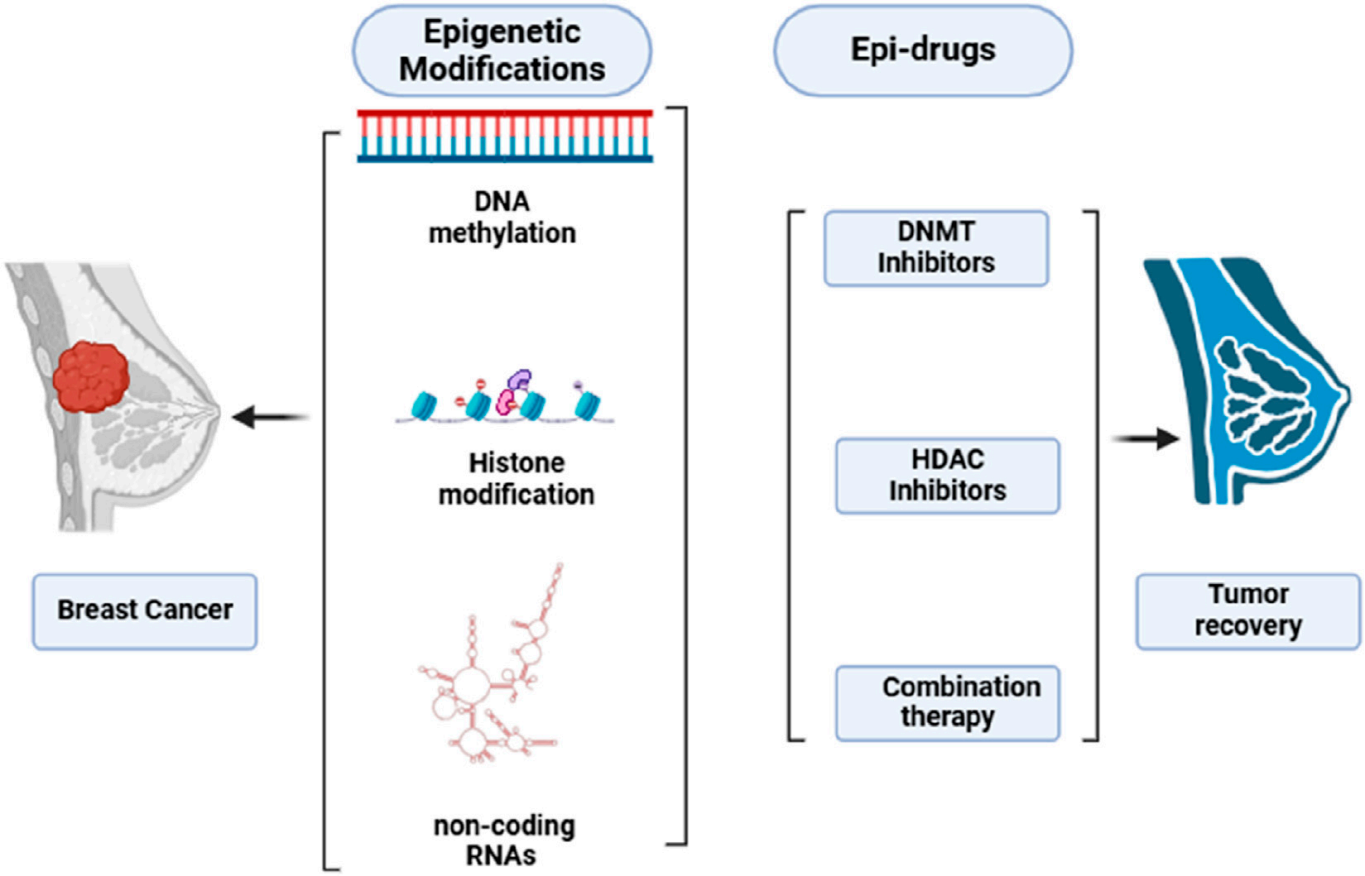
Role of epigenetic modification in progression and epi-drugs in recovery of BC.

Epi-drugs utilized as treatment options have the capacity to trigger the recovery of damaged genes, restore genes that suppress or slow down tumor growth and survival, address the problem of tumor heterogeneity, and prove effective against tumors lacking actionable mutations (Ansari et al., 2016; Kalia, 2015). Additionally, epigenetic medications may have the ability to make cancer cells sensitive again after they have developed resistance to conventional therapy or tyrosine kinase inhibitors (TKIs). Research is currently being conducted on combinations of drugs that address both genetic and epigenetic irregularities, suggesting they could be more effective than those that solely focus on somatic mutations (Kalia, 2015; Brzeziańska et al., 2013). The primary approach for epidrugs focuses on blocking HDACs and DNMTs. An inhibitor of DNMT (DNMTi) positions itself amidst DNA base pairs to prevent the methylation of CpG dinucleotides (Erdmann et al., 2015). DNMTi, a pioneer epidrug, is an analogue of pyrimidine integrated into DNA in replication and triggers the damage response of DNA, leading to cell death. Inhibitors of HDAC (HDACi) reduces Zn2^+^-dependent enzymes activation. HDACi inhibits alterations driven by acetylation of histone and aids in returning to regular state (Kim et al., 2023). Due to the close relationship between cancer instigation and progression with genetic and epigenetic changes, epidrugs represent a promising avenue for developing treatments that target genes involved in cancer epigenetics (Figure 1).

Our comprehensive review of epigenetics in beast cancer offers a current overview and new insights. We conducted a search from its inception until January 2025 for literature on this subject, concentrating on (i) diagnostic and prognostic epigenetic biomarkers in BC, (ii) epigenetic-based therapies for BC, and (iii) clinical trials. We are convinced that advancements in epigenetic research will enhance our comprehension of the disease’s development and foster a more tailored management approach.

## 2 Materials and methods

### 2.1 Search strategy

This research adhered to the 2020 PRISMA guidelines checklist (Table 1) (Page et al., 2021). A search of the literature was carried out in various databases including Google Scholar, PubMed and Scopus. Articles published from the beginning up until January 2025 were considered. To execute a thorough search, following keywords and MeSH terms in various combinations were implemented: (“Breast Cancer”) AND (“diagnosis” OR “prognosis” OR “therapy” OR “DNA methylation” OR “histone modifications” OR “miRNA” OR “lncRNA” OR “TNBC”) (Table 2).

**TABLE 1:** Search terms used for the systematic reviews.

BC epigenetics
Epigenetic diagnosis
BC prognosis
TNBC diagnosis
Epigenetic therapy
BC Epi-drugs
HDAC inhibitors
miRNA prognosis
lncRNA prognosis
DNA methylation prognosis
miRNA diagnosis
lncRNA diagnosis

**TABLE 2:** The most highly investigated epigenetic markers.

S. No.	Potential biomarker	Function	Sample type	Role	References
1.	ALU247	Diagnostic	Plasma	Detect metastatic BC	Agostini et al. (2011)
2.	APC, RARβ	Do	Serum	Early ductal TNBC diagnosis	Swellam et al. (2015)
3.	APC, FOXA1, & RASSF1A	Do	Plasma	Detection and monitoring of BC patients	Salta et al. (2018)
4.	ESR1	Predictive	Peripheral Blood (CTCs)	Predict endocrine therapy efficacy in BC patients	Mastoraki et al. (2018)
5.	FHIT	Diagnostic	Serum	Early diagnosis of ductal BC	Liu et al. (2015)
6.	HYAL2	Do	Peripheral Blood (Leukocyte)	Early diagnose of BC	Yang et al. (2015)
7.	KEAP1	Prognostic & predictive	Tissue	Predict resistance to chemotherapy regimens involving taxanes	Parrella (2018)
8.	RARβ, RASSF1A	Diagnostic	Serum	Detect *in situ* & invasive ductal BC	Kim et al. (2010)
9.	SFN, P16, hMLH1, HOXD13, PCDHGB7 & RASSF1A	Do	Serum	Detection and monitoring of BC	Shan et al. (2016)

### 2.2 Eligibility criteria

To be eligible for inclusion in the review, studies needed to satisfy the criteria as: (i) they must have been carried out in patients with BC or in BC cell lines; (ii) they should focus on diagnostic/prognostic epigenetic biomarkers (iii) studies involving humans must provide relevant clinical pathological characteristics (iv) those with diagnostic and prognostic significance required information regarding survival outcomes; (vi) therapeutic studies and (vii) publications must be in English, and the complete text must be accessible. The search terms are in the Table 1.

Research was excluded if any of the below criteria were met:

(i) Duplicate reports; (ii) studies involving non-human subjects, non-cell line research, animal experimentation, or those not available in English; (iii) studies lacking accessible data or containing partial or retracted information.

### 2.3 Study selection and data extraction

**FIGURE 2:**
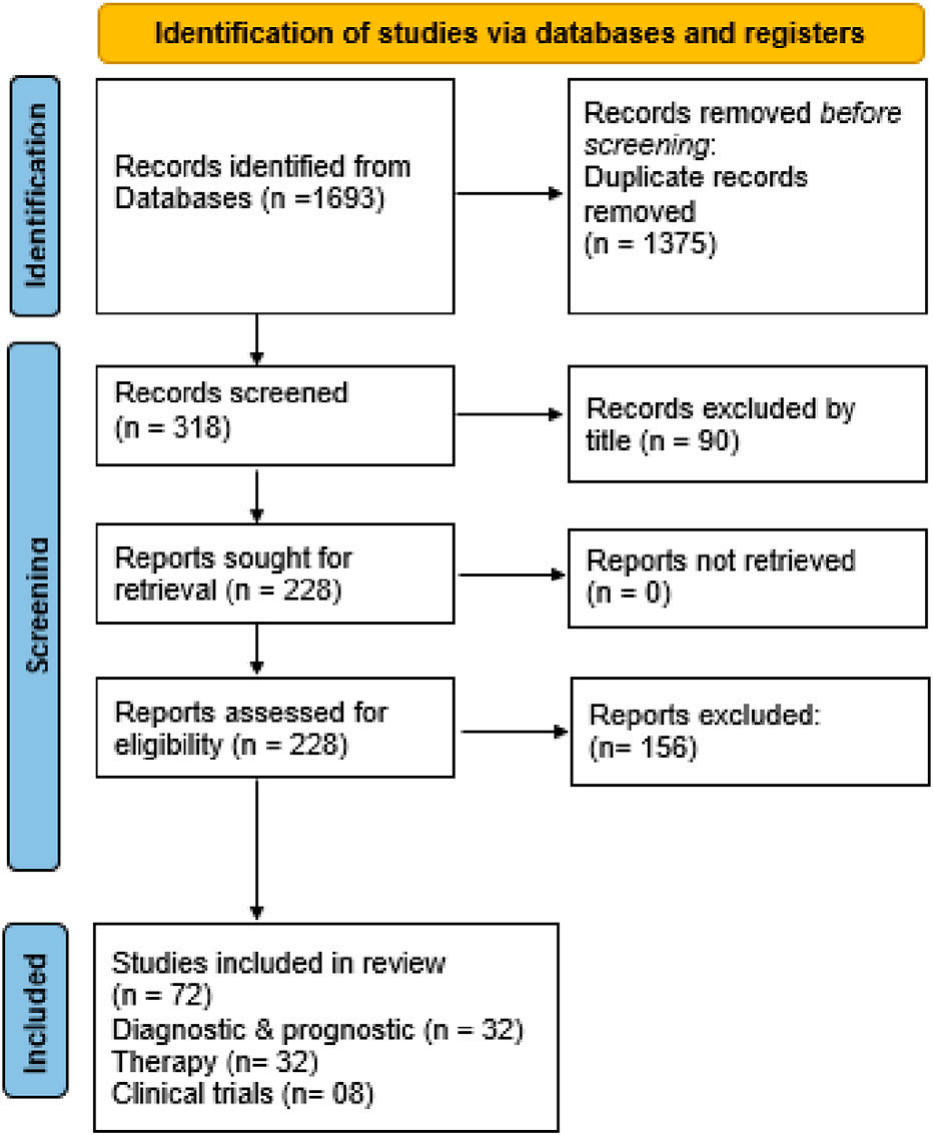
Flowchart showing identification of studies via various databases/registers and included and excluded studies.

The selection process for publications that met the inclusion criteria was conducted manually by the author, without using any automated tools. After eliminating duplicates, a total of 318 items were identified. 90 citations were excluded by title and screened 228 abstracts for retrieval. Finally, 228 eligible studies were included. The queries, along with the corresponding sections of the paper related to diagnosis, prognosis, and therapy, resulted in 72 separate studies utilized across three sections of the paper (Figure 2). Data was gathered from the final 72 studies by the same independent author without the use of automation.

## 3 Results

The search terms employed in this systematic review are summarized in Table 1. The primary three subjects examined are (i) diagnostic and prognostic epigenetic biomarkers in breast cancer; (ii) therapy based on epigenetics; and (iii) clinical trials. After screening, 72 items were selected including, 32 articles for diagnostic and prognostic, 32 for therapy and 08 for clinical trials.

## 4 Detailed results and discussion

### 4.1 Diagnostic and prognostic epigenetic biomarkers

At present, one of the greatest hurdles in oncology is the initial-stage diagnosis of BC (Ginsburg et al., 2020). Delays in treating BC have been linked to more advanced stages of cancer at the time of diagnosis and poorer survival rates (Unger‐Saldaña et al., 2015). While existing diagnostic methods for BC largely rely on ultrasound, magnetic resonance imaging (MRI), mammography, positron emission tomography (PET), computerized tomography and biopsy, these approaches possess several drawbacks, such as high costs, difficulty detecting small tumors, particularly in women with dense breast tissue, time requirements, and unsuitability for younger women. Additionally, the effectiveness of mammography is noted to depend on factors like personal medical history, age, ethnicity, the radiologist’s expertise, and the excellence of the technique used (Wang, 2017). Consequently, there is dire need to develop a sensitive and rapid diagnostic method for early-stage BC to enhance existing diagnostic options. Recently, advancements in computational and analytical methods have led researchers to focus on the early detection of BC through the creation of specific biomarkers. Thus, discovering new diagnostic and prognostic biomarkers is essential for early identification of BC and will offer improved possibilities for its prevention and treatment, ultimately aiming to significantly lower mortality and morbidity rates associated with BC globally (Sarvari et al., 2022).

The significance of identifying biomarkers extends beyond their prognostic role, which indicates the likely progression of a disease; they also offer insights into how a patient may respond to a chosen treatment. Consequently, it is essential to have diagnostic biomarkers for screening and categorizing breast cancer patients. Conversely, prognostic biomarkers are crucial for estimating a patient’s survival prospects (Louie et al., 2021). While previous research on biomarkers has largely concentrated on non-epigenetics, current studies have investigated the potential of epigenetic markers in solid as well as liquid biopsies from BC patients (Alba-Bernal et al., 2020).

Epigenetic changes, like irregular methylation of DNA and acetylation of histone at gene promoter regions, represent some of the earliest events in the mechanism that leads to cancer, as they play a role in silencing specific genes. Reports indicate that the quantity of genes with irregular methylation detected in BC is rising quickly (Zubor et al., 2019). Abnormal methylation of DNA is a key biomarker for analysis in liquid biopsies because of several factors: its early appearance, cancer-specific characteristics, organic stability, and presence in body fluids. Its relatively high stability and detectability in circulating cell-free tumor DNA (ccfDNA) from liquid biopsies provide the opportunity to use DNA methylation as a quick, dependable, cost-effective, and non-invasive method for testing breast cancer (Salta et al., 2018; Egger, 2018). It is clearly established that abnormal DNA methylation is a significant factor in development of BC and its resistance to treatment. Additionally, research indicates that changes in the DNA methylation pattern in blood of BC patients occur several years prior to the clinical diagnosis of the disease (Xu et al., 2020). As a result, abnormal DNA methylation may serve as a significant biomarker for BC (Cheng et al., 2019).

**FIGURE 3:**
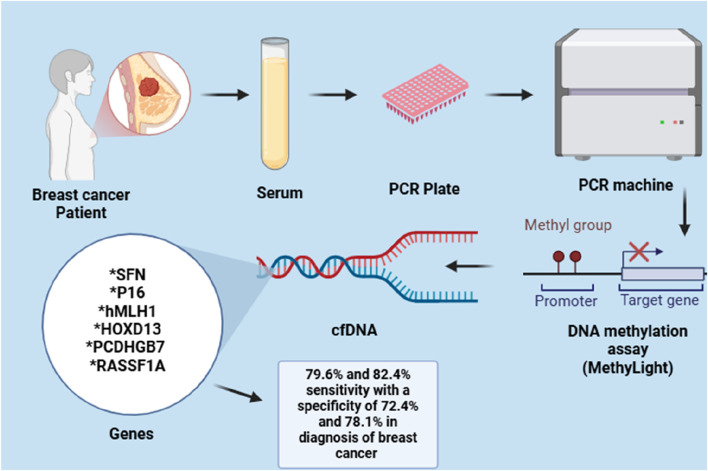
Methylation detection in a six-gene panel for BC diagnosis.

For example, the hypermethylation of hyaluronoglucosaminidase 2 (HYAL2) in blood can be identified in the initial stages of breast cancer (BC) cases. This indicates that the methylation level of HYAL2 may serve as an early indicator for detecting BC, demonstrating a high sensitivity of 64% and a specificity of 90% (Yang et al., 2015). Due to the heterogeneity observed both within and between tumours of BC, it has been suggested that relying on a single epigenetic biomarker for BC detection may be effective for one subtype but not for others, potentially resulting in incorrect diagnoses. As a result, several gene panels have been created and assessed to enhance detection sensitivity of BC. Like, Kim et al. investigated a panel of two gene, RARβ and RASSF1A, with 94.1% and 88.8% notable specificity and sensitivity for BC detection, respectively (Kim et al., 2010). In a different study, a panel of seven methylated genes, including BRCA1, APC, CCND2, SCGB3A1, FOXA1, RASSF1A and PSAT1, was able to identify BC with great specificity and sensitivity with 95.55% accuracy. In a similar manner, a gene panel with six methylated genes (RASSF1A, SFN, P16, PCDHGB7, HOXD13 and hMLH1) and a three-gene panel (RASSF1A, APC and FOXA1) demonstrated a high sensitivity and specificity in detecting BC in serum, as depicted in the Figure 3 (Shan et al., 2016).

Furthermore, new DNA methylation markers, including PRAC2, TDR10, and TMEM132C, were discovered to be promising diagnostic and prognostic indicators because of their elevated expression in breast tumor tissue, particularly among ER^+^ patients (de Almeida et al., 2019). Additionally, Nandy et al. suggested utilizing five-panel histone epigenetic biomarkers (ɣH2AX, MacroH2A.1, APLF, H2Bub1 and HJURP) that may act as a possible prognostic indicator for assessing likelihood of BC metastasis (Nandy et al., 2020).

The epigenetic features of BC can likewise be identified through ctDNA analysis for the purpose of timely detection and targeted treatment of BC (Rohanizadegan, 2018). Agostini and his team found the ALU247 methylation in BC by employing the MethyLight^®^ technique, achieving over 69% specificity and 99% sensitivity (Agostini et al., 2011). Liu and colleagues investigated the extent of FHIT promoter methylation and found a significant association with ductal breast carcinoma; this could be beneficial for initial detection of this form of BC (Liu et al., 2015).

Epigenetic biomarkers may serve as indicators to anticipate how patients will respond to therapeutic drugs (Berdasco and Esteller, 2019). Instances comprise of KEAP1 gene methylation, which has been associated with improved overall survival; this could act as a biomarker indicating resistance to chemotherapy treatments that include taxanes (Parrella, 2018). Research indicates that hypermethylation of the p16 promoter in breast cancer suggests that p16 could serve as a prognostic and predictive marker for assessing hormonal therapy response (Goyal et al., 2019). In addition, hypermethylation of p16 is notably associated with a proposed hypermethylation profile indicative of pre-cancerous changes, which includes RARβ2, ERα, BRCA1 and BRCA2. This implies that the hypermethylation of the p16 promoter in these genes may be identifiable in the initial stages prior to any pathological alterations; thus, it could serve as a means to identify women who require close surveillance for breast cancer (Thomas and Marcato, 2018).

Methylation of ESR1 DNA in plasma ctDNA samples is expressively linked with absence of estrogen receptor (ER) expression in excised tumors, which is related with a reduced response to endocrine therapy (Martínez-Galán, 2014). As a result, ESR1 could act as a possible prognostic biomarker for the effectiveness of endocrine therapy (Stone et al., 2015; Mastoraki et al., 2018). The link between BRCA1 hypermethylation and heightened sensitivity to platinum-based chemotherapy in ovarian and BC can also be leveraged to use BRCA1 as a predictive biomarker for treatment response to platinum-based chemotherapy in BC (Liu et al., 2015; Laham-Karam et al., 2020).

FDA has approved a blood-based test for biomarkers related to breast cancer. Various cancer antigen biomarkers, including CA15−3, CA27.29, CA-125, CEA (carcinoembryonic antigen), and CTCs, are primarily suggested as prognostic indicators to evaluate the effectiveness of treatment and detect disease recurrence, rather than for early diagnosis. At present, the only screening test utilized for hereditary BC involves mutation analysis applying gene mutation markers (BRCA1 and BRCA2) (Tang et al., 2016). Furthermore, molecular *in vitro* diagnostics (IVDs) currently employed include tumor screening instruments like endopredict, mammaprint, prosigna and oncotype DX which utilize gene mutational and expression profiles obtained from traditional tissue biopsies rather than from methylation of DNA. Oncotype DX is widely utilized and is specifically intended for patients with ER^+^/HER2- status and no lymph node involvement in primary BC. EndoPredict is a novel prognostic tool that assesses expression of eight specific genes to evaluate recurrence risk in BC patients who are ER^+^/HER2- (Vieira and Schmitt, 2018). These cancer-screening tools were created with the goal of classifying breast cancer patients into different risk and treatment categories to aid in making decisions about adjuvant therapy. Nonetheless, their effectiveness in clinical settings is confined to specific subtypes of breast cancer, which limits their practical application. IvyGene is a recognized test in the USA that uses DNA methylation to identify the early stages of four prevalent cancers: lung, colon, breast and liver. By leveraging a panel of 46 biomarkers, it can assess the likelihood of these cancers through blood samples from patients suspected of having cancer (Sher et al., 2022).

Recently, Garcia and colleagues suggested that cfDNAs found in plasma could serve as a prognostic indicator for individuals with metastatic BC (Fernandez-Garcia et al., 2019). Furthermore, it was demonstrated that cfDNA level and CTCs count serve as indicators of overall survival. On other hand, levels of cfDNA stand as exclusive prognosticator for progression-free survival (PFS) and for distinguishing between treatment responders and non-responders. Moreover, the authors indicated that evaluating cfDNA and CTCs yields more insightful information than combination of two traditional biomarkers (AP and CA15-3) in forecasting OS. A recent investigation also explored the predictive value of blood circulating cell-free DNA (ccfDNA) in both early and advanced stages of BC (Panagopoulou et al., 2019). The research included three groups of patients and one group of healthy controls, comprising 150 breast cancer patients undergoing adjuvant therapy and 16 patients receiving neoadjuvant therapy, along with 34 individuals diagnosed with metastatic breast cancer and 35 healthy participants. The results indicated that higher levels of blood ccfDNA were significantly related to mortality rates, reduced progression-free survival (PFS), and lack of response to treatment in the metastatic breast cancer group, but not in the other groups. Notably, the study identified three types of ccfDNA fragments based on their origin: those from apoptosis (∼160 bp), necrosis (greater than 10,000 bp), and active secretion from viable cells (2000 bp), which were examined through size profiling via capillary electrophoresis. Patients exhibiting a higher tumor load in metastatic and neoadjuvant groups typically displayed an abundance of tinier fragments and a more fragmented distribution pattern. In addition, methylation status of five cancer-related genes—KLK10, SOX17, GATA3, WNT5A and MSH2 has also been assessed in plasma ccfDNA of BC patients. The findings indicate that methylation of KLK10, WNT5A, SOX17 or the concurrent methylation of at least three genes occurred more often. Furthermore, statistically a significant relationship was also found between methylation of WNT5A and larger tumor size as well as poor prognostic indicators in advanced stage disease, which correlated with shorter overall survival. In metastatic cohort, methylation of SOX17 was notably linked to higher mortality rates and reduced progression-free survival (PFS) and OS. Additionally, MSH2 methylation was observed more recurrently in adjuvant and metastatic groups, while GATA3 methylation was more prevalent in neoadjuvant group. Ultimately, the researchers concluded that circulating cell-free DNA (ccfDNA) represents a highly effective predictive classifier for metastatic BC when combined with established clinicopathological factors, which may assist in achieving timely and precise diagnosis and prognosis (Peled et al., 2020).

### 4.2 Epigenetic based therapy for BC

Histone methyltransferases alteration promote development of epidrugs aimed at various chromatin regulators (Richart and Margueron, 2020). Epigenetic agents can prompt cell death in response to hormone therapy. Treatment with tamoxifen triggers autophagy, resulting in increased cancer cell mortality. Nevertheless, it may also lead to the development of tamoxifen-resistant BC. HDAC inhibitors can enhance pro-apoptotic proteins such as BAK and BAX expression. Consequently, they may be utilized in conjunction with tamoxifen, primarily steering ER^+^ BC cells towards apoptosis. This approach could pave the way for improved clinical trials involving a combination of HDACi, exemestane, and tamoxifen (Kim et al., 2023). Luminal-B BC are characterized as ER^+^ and can be either HER2^+^ or negative subtypes. The clinical effectiveness of iadademstat as an anti- SOX-2 epigenetic therapy for BC has shown promise in targeting cancer stem cells driven by SOX-2, particularly in the SOX-2-abundant luminal-B HER2+ category (Fang et al., 2021). Patients with luminal-B BC using iadademstat led to a notable decrease in SOX2 expression, indicating a focused approach to targeting SOX2-driven cancer stem cells (CSCs). Epigenetic strategies targeting LSD1, such as iadademstat, show considerable promise for the prevention of breast and ovarian cancers in cancer therapy (Cuyàs et al., 2020).

#### 4.2.1 DNMT inhibitors (DNMTis)

DNMT inhibitors hold significant promise for the treatment of cancer. Recently, their application has been highlighted within the realm of immuno-oncology. DNMT inhibitors enhance the immunogenicity of tumors by promoting the secretion of cytokines through immune cell activation. DNMT inhibitors represent a hopeful therapeutic option for cancer treatment and demonstrate anti-tumor effects specifically against BC (Wong, 2021). Yanrong Su and colleagues explored potential of using DNMT inhibitors to target epithelial-mesenchymal transition (EMT) for treatment of TNBC. It was demonstrated that extremely aggressive TNBC cells could be reprogrammed into less aggressive cells through the process of EMT. Additionally, DNMTi displayed antitumor properties by hindering cell proliferation, including the induction of cell cycle arrest. These findings indicate that DNMTi may serve as auspicious therapeutic entity with antitumor effects (Su et al., 2018). Decitabine (5-aza-2′-deoxycytidine), a drug that demethylates DNA, is a DNMT inhibitor approved by the FDA. Decitabine binds irreversibly to DNMTs and interacts with enzymes on DNA, leading to the failure of DNMT function. Its effectiveness in treating hematological cancers has been well established. In the case of BC, TNBC treated with decitabine demonstrated a high level of sensitivity. This response not only resulted in the degradation of DNMT1 through a proteasomal-dependent mechanism but also led to the degradation of DNMT3A and DNMT3B in lower concentrations. Additionally, patient-derived xenograft (PDX) organoids have exhibited significant tumor growth inhibition properties when exposed to low doses of decitabine (Yu et al., 2018). Additional research showed that decitabine triggered autophagy in BC cells, as evidenced by a rise in autophagy marker LCB-II (Vernier et al., 2020).

Azacitidine may work as a new therapeutic option in treatment of preclinical BC. Treatment with azacitidine hinders the metastasis of breast cancer to the brain. It reduces Wnt signaling pathway, as well as cell invasion, cell migration and tumour development in cells that colonize the brain (Butler et al., 2020). In a separate study, the use of a combination therapy involving azacitidine and vorinostat led to an increase in the expression of PD-L1 mRNA (Terranova-Barberio et al., 2017). Guadecitabine has been proposed as a substitute for traditional DNMT inhibitors, like decitabine and azacitidine, due to its potential for use in first-line therapy. Guadecitabine enhances the expression of PD-L1 and MHC class I while reducing the proliferation of tumor cells. Additionally, early administration of guadecitabine prevents the initiation of tumor growth *in vivo* (Gilmer, 2020). Furthermore, when combined with HDAC inhibitors, guadecitabine has the ability to reprogram aggressive triple-negative breast cancer (TNBC) cells. Additionally, guadecitabine enhances the expression of MHC class I and II in TNBC (Wong, 2021). As a result, guadecitabine could serve as an effective anti-tumor medication for individuals diagnosed with BC.

Liraglutide, an anti-diabetic, can function as a DNMT inhibitor in BC both in laboratory settings and in Ehnlich mouse tumor models. The findings indicated that it decreased cell migration, viability and DNMT activity (Ayipo et al., 2022). It effectively enhances anti-proliferative effects while impairing migration and movement of mesenchymal BC cell lines. Notably, in contrast to earlier medications, hypermethylation of the DNMT gene was not found following treatment with liraglutide. The use of half-dosages of chemotherapeutic agents alongside liraglutide greatly minimizes side effects, including toxicity and reproductive issues (Chequin et al., 2021). Hence, liraglutide may be assessed as a novel adjunct to enhance BC therapy.

#### 4.2.2 HDAC inhibitors

HDACs have a crucial role in regulating important genes related with the development and survival of cancer cells. Consequently, inhibiting HDACs is an effective approach for treating cancer cells. HDAC inhibitors can be categorized into benzamides, short-chain fatty acids, hydroxamic acids (hydroxamates), sirtuin inhibitors and cyclic tetrapeptides (Cappellacci et al., 2020). In clinical trials for cancer, it is typical to use combination therapy that includes HDAC inhibitors.

Vorinostat was the inaugural HDAC inhibitor to receive approval from FDA (Zhou et al., 2019). Vorinostat reduces growth of TNBC cells by enhancing the expression of miRNAs that in turn activates tumor suppressor genes. Furthermore, when vorinostat is used alongside simvastatin (a medication used to lower cholesterol), it can trigger apoptosis by disrupting Rab7 prenylation and inhibiting the fusion of autophagosomes and lysosomes in TNBC (Patra et al., 2022). Combination therapy demonstrated apoptotic effects as well via blocking Rab7 prenylation *in vivo* in xenograft mice. This suggests that Rab7 is a potential drug target for combined use of simvastatin and vorinostat. In another investigation, pairing of letrozole (an aromatase inhibitor) and vorinostat resulted in decreased BC cell spread, induction of apoptosis, and the differentiation of peripheral blood mononuclear cells into osteoclasts. This blend might reduce risk of osteoporosis among BC patients. Moreover, administering vorinostat in conjunction with immune checkpoint inhibitors, such as PD-1 and CTLA-4, can promote tumor apoptosis and shrinkage in TNBC (Zucchetti et al., 2019).

Transwell assays for migration, invasion and healing of wound have demonstrated that treatment with trichostatin A (TSA) significantly hampers the invasion and migration capabilities of BC cells (Wang et al., 2020). TSA, a strong inhibitor of pandeacetylase, has the ability to influence the transcriptional function of ERβ in BC that does not express ERα, leading to a response in hormone receptor-negative BC cells to tamoxifen. This increases the susceptibility of TNBC cells to tamoxifen effects (Wang et al., 2021). Panobinostat, an HDAC inhibitor has the ability to enhance acetylation of histone, influencing cell cycle, and promote apoptosis in BC. It has suppressed proliferation and increased acetylation of histone in TNBC cells in an *in vivo* study (Qin et al., 2019). Panobinostat has the ability to reactivate silenced ERα in TNBC and enhance tamoxifen responsiveness. Therefore, pairing of trastuzumab and panobinostat has been proposed as a treatment for HER2-positive cases (Laengle et al., 2020). A different combination therapy involving letrozole and panobinostat reduced expression of aromatase in BC cells that are hormone-responsive, indicating that such combination treatments are probably effective against hormone receptor-positive/aromatase-positive breast cancer (Huang et al., 2019). Varprobic acid (VPA) has newly been recognized as a potential therapy for cancer. For more than five decades, it has effectively been utilized to manage schizophrenia, bipolar disorder and epilepsy (Wawruszak et al., 2021). VPA demonstrates strong antitumor activity both in laboratory settings and in animal models, whether used individually or together with demethylating cytotoxic agents, leading to positive results in clinical trials. VPA suppresses the proliferation of HER2+ BC cells by enhancing p21 WAF1 expression (Wawruszak et al., 2021). VPA also triggers apoptosis and promotes acetylation of histone H3 by disrupting hsp90 function (Wu et al., 2021). VPA in conjunction with 5-aza-2′-deoxycytidine can trigger RA receptor β2 tumor suppressor gene’s transcription reactivation in BC, resulting in increased apoptosis (Ediriweera et al., 2019).

HDACi effectiveness in solid tumours as a standalone treatment is not consistently positive. For this reason, it is advised to use them in combination with other therapies like hormone therapy, chemotherapy and immunomodulatory agents. HDAC inhibitors have the ability to restore proteins unchecked acetylation linked to the pathways of cancer and reactive tumor suppressor genes, which can result in the arrest of the cell cycle and induce apoptosis in cancer cells (Song et al., 2021). Resistance to HDAC inhibitors (HDACi) poses a significant barrier to effective treatment with these agents. Combining therapies has demonstrated much stronger effects than using HDACi alone, improving their clinical effectiveness. Ideal combination treatments can effectively address the issue of HDACi resistance. The discovery of new selective HDAC inhibitors, along with identifying predictive biomarkers for their use, and a deeper understanding of the mechanisms behind HDACi will enhance their application in breast cancer therapy (Kim et al., 2023).

In a recent investigation, researchers successfully developed patient-derived organoids for TNBC, creating a valuable model of conducting high-throughput drug screenings. Through this approach, they discovered new epigenetic compounds that target histone deacetylase, JAK/STAT, aurora kinase and histone demethylases pathways, demonstrating a notable tumour-killing properties against TNBC. In the identified compounds, TAK-901, panobinostat, JIB-04 and pacritinib displayed greater effectiveness compared to paclitaxel. These results emphasize that these compounds may serve as good therapeutic options for TNBC and support the importance of patient-derived organoids in progressing drug discovery (Rao et al., 2024).

#### 4.2.3 Bromodomain and extraterminal domain (BET) inhibitors

In the treatment of malignant tumors, bromodomain (BRDs) and extraterminal (BET) proteins inhibitors have been appeared to be novel targeted medicines that control the epigenetic alterations in such conditions (Filippakopoulos et al., 2010). BRDs constitutes a family of proteins referred to as BET family of four members, namely, BRDT, BRD2, BRD3 and BRD4 (Filippakopoulos et al., 2012). The most potent and extensively studied BET protein is BRD4, also referred to as “reader” of lysine acetylation (Dawson et al., 2011). This BRD4 protein is a crucial transcriptional regulator and is involved in regulation of gene expression for a number of super-enhancer associated genes, including the well-known oncogene c-MYC (Bell et al., 2019). This implies that modifying proteins of BET family may be a promising cancer treatment approach. A number of BET inhibitors, such as JQ1, is thought to be a pan-BET inhibitor that has comparable suppressing impacts against BD1 and BD2, were among the first BET inhibitors to be made available (Filippakopoulos et al., 2010). Among 41 human BD-containing proteins, JQ1 possessed the greatest affinity for binding to BRD4 (Filippakopoulos et al., 2010). BET inhibitors are innovative targeted medicines that control epigenetic alterations in the treatment of malignancies. These inhibitors may block the over expression of oncogenes, making them as possible cancer treating agents.

A small molecule inhibitor of BRD2, BRD3 and BRD4, OTX015, having a structure similarity to JQ1, represents a significant advancement in its oral administration (Boi et al., 2015). Although the BRD3 expression remained constant, exposure to OTX015 resulted in a significant reduction in BRD2, BRD4, and c-MYC as well as a spike in HEXIM1 protein levels (Coudé et al., 2015). These alterations imply that OTX015 might cause growth inhibition, apoptosis, and cell cycle arrest (Baratta et al., 2015). Moreover, *in vitro* studies have demonstrated that OTX015 exhibits encouraging synergistic effects with a number of anticancer medications, namely BTK and mTOR inhibitors (Boi et al., 2015). BRD2, BRD2 BRD3 and BRD4 are mainly targeted by I-BET762, which is a pan-affinity oral BET inhibitor (Piha- et al., 2020). The study reports that I-BET762 primarily acts by down regulating MYC and IRF4, as well as upregulating HEXIM1 (Zhang G. et al., 2015). NHWD-870 is a newly developed BET inhibitor that exhibits well-known efficacy in preventing the growth of various cancers by suppressing the expression of macrophage CSF1 in tumor cells. It is evident from a cytometric assay that NHWD-870 is more potent than three well-known BET inhibitors in clinical studies, such as GSK525762, BMS-986158 and OTX015 in order to treat multiple cancer types (CXHL200250), NHWD-870 is currently undergoing Phase I clinical trials (Zhang G. et al., 2015). Recent studies have shown that NHWD-870 significantly reduces metastasis and melanoma invasion both *in vivo* and *in vitro* by controlling SPINK6, (Hu et al., 2023). Further, on melanoma, NHWD870 and cytarabine showed synergistic effects both *in vitro* and *in vivo* (Deng et al., 2022).

#### 4.2.4 Non-coding RNA-Based therapies

According to data from the Human Genome Project, more than 90% of genome is transcribed but around about 2% of it is translated while the total RNAs consisted of 98% non-coding (nc) RNAs. In contrast to genes of protein-coding, research has demonstrated that ncRNAs have important functions in many biological processes, including transcription, chromatin remodeling, signal transduction and post-transcriptional modification (Merrill et al., 2020); ncRNA activity is likely responsible for the abnormal expression levels of several genes associated with the onset of BC (Wang et al., 2019). Moreover, ncRNAs may be targeted therapeutically, and their delivery can be based on current framework of oligonucleotide delivery and RNAi interference in targeting mRNAs that code for proteins (Levin, 2019). As a result, knowing specific ncRNA signatures can aid in the comprehension of intricate BC cellular mechanisms and promote advancements in the diagnosis and management of BC subcategories. The well-researched small microRNAs (miRNAs) and long non-coding RNAs (lncRNAs) are the two main types of non-coding RNAs. While miRNA comprises 19–28 nucleotides, lncRNA often has more than 200 (Yardim-Akaydin et al., 2022). Argonaute proteins (Ago proteins) communicate with small RNA species in typical sense, such as miRNAs, piwi-interacting RNAs (piRNAs), and small interfering RNAs (siRNAs), thereby mediating RNA silencing effects (Xiong et al., 2023). On the other hand, by controlling production of miRNAs or transcription factors, lncRNAs can either favorably or unfavorably affect the characteristics of BC cell proliferation, invasion, metastasis, and stemness (Kopp and Mendell, 2018). The disruption in both transcript types’ is frequently associated with every cancer that has been investigated so far, which has significant impact on all of the main characteristics of cancer. Numerous RNA-based therapies have emerged, such as CRISPR-Cas9-based gene editing, miRNA mimics, miRNA sponges, therapeutic circular RNAs (circRNAs), antisense oligonucleotides (ASOs), siRNAs, short hairpin RNAs (shRNAs), ASO-anti-microRNAs (antimiRs), and miRNA mimics. These medications are described in a number of excellent reviews (Winkle et al., 2021). It is possible to use ncRNAs as possible therapeutic targets in BC treatment by expressing specific tumor-suppressing mRNAs to manage BC or by developing tailored siRNAs or miRNAs to prevent tumor-promoting ncRNAs. The use of nanodrug delivery systems in BC treatment has also demonstrated prodigious promise, with great nanodelivery/nanoparticle-based strategies developed by employing various molecules for systemic drug delivery and increased targeted delivery of tumor ncRNAs with minimum side effects.

Given the possibility of ncRNA therapies, the associated challenges of complexity and diversity, stability problems, delivery methods, specificity, further investigation are necessary to produce clinically useful applications. Illuminating the various roles and modes of action of ncRNA is one of the biggest difficulties in the field of present time research. This is important because it will help determine its clinical relevance and develop its potential application as a therapeutic target or biomarker (Nemeth et al., 2024).

##### 4.2.4.1 lncRNA therapy in breast cancer chemoresistance

Metastasis and resistance are the two main issues that emerge during the treatment of breast cancer (Echeverria et al., 2019). LncRNAs interact with many RNAs and proteins to affect drug resistance and are dysregulated in a variety of cancers. Aromatase inhibitors (AI) have been shown to upregulate the lncRNA DIO3OS in patients with breast cancer. DIO3OS works by stabilizing the mRNA for lactate dehydrogenase A (LDHA) through its interaction with polypyrimidine bundle-binding protein 1 (PTBP1), which increases the expression of LDHA and facilitates glycolytic metabolism. In other words, DIO3OS gives AI-resistant cells a growth advantage by controlling the splicing switch to increase aerobic glycolysis. As a result, LDHA activity inhibition using DIO3OS knockdown techniques may re-sensitize breast tumor cells to chemotherapies (paclitaxel) or anti-HER2 treatments (trastuzumab) as a target for BC treatment (Chen et al., 2022). In a study aimed at treating paclitaxel-resistant BC, it was shown that LINC00115 activated the HIF1α signaling pathway by acting as an RNA linker that recruited the SETDB1/PLK3 complex and was highly increased in paclitaxel-resistant BCSC (Luo et al., 2024). As an oncogene in breast cancer, SETDB1 is crucial in treating resistance to endocrine therapy (Liu Z. et al., 2022). By blocking its ubiquitinated breakdown pathway, methylation of PLK3 results in the failure of HIF1α phosphorylation, which increases the stability of the HIF1α protein. In consequence, HIF1 can improve LINC00115 stability, which in turn improves BCSC properties and encourages chemotherapy resistance and metastasis in BC. Therefore, in an animal xenograft model of BC metastasis, SETDB1 inhibitors in conjunction with LINC00115 suppression markedly increased the effectiveness of paclitaxel treatment. Chen et al. (2023) discovered that LINC02568 competitively binds miR-1233-5p to the estrogen receptor ESR1 mRNA itself, trans-regulating the stability of ESR1 mRNA and controlling the transcriptional activation of target genes in the cytoplasm induced by estrogen and estrogen receptors. Through cis regulation in the nucleus, LINC02568 contributes to the transcriptional activation of nearby genes CA12, helping to maintain a particular pH both inside and outside tumor cells. Through cis-regulation, LINC02568 contributes to the transcriptional activation of the nearby gene CA12 in the nucleus, which in turn helps to maintain a particular pH both outside and inside the tumor cell. Tamoxifen-resistant cells of BC were once again sensitive to tamoxifen after ASO targeting LINC02568 dramatically reduced the development and tumor growth of estrogen receptor-positive BC cells. As a result, endocrine medications or CA12 inhibitors work in cooperation with ASO targeting LINC02568 to limit tumor growth. Doxorubicin-resistant tumor cells were shown to have significantly higher levels of LINC00460. LINC00460 and FUS work together to influence the effectiveness of intron removal during mRNA maturation, which in turn enhances MYC expression. On the other hand, c-MYC directly activates the transcription of LINC00460, creating a favorable feedback cycle in BC cells that increases resistance to tamoxifen. The simultaneous c-MYC suppression and LINC00460 depletion significantly re-sensitized ADR cells towards doxorubicin. Accordingly, Yang et al. (2024) proposed the simultaneous antagonism of c-MYC and LINC00460, which most likely effectively eliminated the positive feedback loop and might be a promising new strategy to enhance treatment outcomes for patients who have developed acquired resistance to doxorubicin treatments. Liu X. et al. (2022) discovered that lncRNA aspartate-trna synthetase-antisense RNA 1 (DARS-AS1) was overexpressed in TNBC and that its silence successfully prevented tumor development and liver metastasis in another investigation of adriamycin resistance in BC. They developed EXOs-CL4, a natural nanomedicine delivery system tailored to TNBC, and loaded it with DARS-AS1 siRNA and DOX (DARS-AS1 siRNA/DOX@EXOs-CL4), which together prevented tumor growth, metastasis, and anti-apoptotic effects (Liu et al., 2023). In order to overcome chemotherapy resistance in patients with breast cancer, resistance-causing lncRNAs can be utilized to create new targeted and customized therapies. This offers a fresh strategy for implementing potential individualized treatment modalities.

##### 4.2.4.2 miRNA-targeted therapy in breast cancer

miRNAs produced from circulating extracellular vesicles (EVs) have become increasingly recognized as next-generation “therapeutic diagnostic” tools for cancer that have significant clinical utility (Giordano et al., 2023). Exosomes serve as messengers between tumoral and stromal cells, facilitating the transfer of miRNAs from donor cells to neighboring cells (Donnarumma et al., 2017). The full miRNA cargo, which includes pre-miRNAs and proteins that participate in miRNA biogenesis and function, including RISC loading complex (RLC), Dicer, trans-activating response element RNA-binding protein (TRBP), and AGO2, is present in exosomes derived from cancer cells. As a result, pre-miRNAs can be utilized to yield mature miRNAs (Melo et al., 2014). Through receptor-ligand contact, exosomes carrying miRNA are absorbed and continue on to control recipient cells’ gene expression (Zhang J. et al., 2015). Numerous pathways linked to cancer include ubiquitination, one of the most common and significant post-translational modifications (Wang et al., 2018). Through their regulation of ubiquitination, deubiquitinating enzymes (DUB) play a role in cancer regulatory pathways. It was demonstrated that MDA-MB-231 and MCF7 cells treated with exosomes generated from cancer-associated fibroblasts (CAF) had significant expression of miR-500a-5p. MiR-500a-5p was also found to be upregulated in CAF and exosomes produced from CAF. MiR-500a-5p is transported from CAF to cancer cells, where it binds to ubiquitin-specific peptidase 28 (USP28) to stimulate growth and metastasis. By sponging USP28, MiR-500a-5p encourages the growth and spread of breast cancer (Chen et al., 2021). By targeting Dickkopf 3 (DKK3) and NUMB, (Yang et al., 2021), it was found that exosomes generated by BC cells upon stimulation with DOX or PTX transported miR-378a-3p and miR-378d to nearby cells, activating the WNT and NOTCH stemness pathways and causing resistance. Furthermore, chemotherapy increased the levels of miR-378a-3p and miR-378d in cells and exosomes by activating the EZH2/STAT3 pathway in tumor cells. More significantly, in a tumor xenograft model in nude mice, the combination of chemotherapeutic drugs with the EZH2 inhibitor tazemetostat reversed chemotherapy-induced exosome-induced resistance. Additionally, exosomes released by BC cells transmit miR-148-3p, miR-520b, and miR-138-5p to target macrophages to activate M2 polarization, hence increasing tumor growth. Therefore, antagonist tumor suppressor miRNAs can be delivered via exosomes in cancer treatment. Inhibiting cancer can also be achieved by blocking exosomes from entering the blood stream or by stopping target cells from fusing or absorbing exosomes. In order to cure cancer, it can also be removed from the patient’s circulatory system, altered, and transferred to the same patient (Wortzel et al., 2019). Synthetic oligonucleotides are miRNA antagonists (antagomiRs), which target and inhibit oncogenic miRNAs with comparable lengths. Synthetic nucleotide structures called miRNA sponges function in a similar way to antagomiRs in that they bind to oncogenic miRNAs within cells and disrupt their activity. Reduced cell growth, migration, and invasion were the outcomes of transfecting metastasis-associated miRNA-10b overexpressing MDA-MB-231 cells with miRNA-10b-sponges. It has been shown that miRNA-10b overturning by miRNA-10b-sponges upregulates HOXD10, which prevents BC metastasis (Liang et al., 2016). Both tamoxifen-resistant and chronically estrogen-deficient MCF7 cells showed downregulated miR-378 (Ikeda et al., 2015). Arabkari et al. discovered that XBP1, a transcription factor, could suppress the expression of miR-378 and PARGC1B, the host gene for miR-378, during UPR, a cellular stress response pathway that maintains protein homeostasis in the endoplasmic reticulum. MiR-378 is growth inhibitory in ER^+^ BC. Consequently, they developed ORIN1001, an IRE1 inhibitor that prevents the production of XBP1, which is currently undergoing a phase 1 trial to assess its effectiveness in patients with recurrent resistant metastatic BC or advanced solid tumors (Arabkari et al., 2023).

##### 4.2.4.3 Targeted delivery during miRNA therapy

The capacity of tumor cells to undergo EMT, invasion, and metastasis may be diminished by over expression of some miRNAs that function as oncogenes in malignancies. But the biggest obstacle to using miRNAs as therapeutic agents is still their distribution within cells (Mollaei et al., 2019). Compared to normal breast cells, BC cells have been shown to have a significant downregulation of miR-206 levels that target NOTCH (Adams et al., 2009). Using upregulation of miR-206 mimics by gold nanocomplexes, Chaudhari et al. (2022) demonstrated decreased expression of NOTCH. Additionally, miR-206 delivered via gold nanocomplexes in MCF-7 cells was able to modify mitochondrial membrane potential, cause G0-G1 cell arrest, and prevent cell growth. Garrido-Cano et al. (2023) administered miR-200c-3p for breast cancer treatment using mesoporous silica nanoparticles. By down regulating ZEB1 and ZEB2, the well-known tumor suppressor miRNA miR-200c-3p prevents BC tumor growth and metastasis. They show that miR-200c-3p-loaded nanoparticles are safe and efficient method for delivering miRNAs to specific tumors and a promising approach for BC treatment. Another study discovered that breast tumor cells not only transmit oncogenic miRNA factors, but also cause expression of miRNA (miR-182) in macrophages. Additionally, miR-182 supports selective activation of macrophages to drive the formation of tumors. Additionally, they discovered that employing cationic mannan-modified extracellular vesicles to load miR-182 inhibitors and delivering the inhibitors precisely into macrophages successfully prevented the growth of breast tumors and reduced macrophage alternative activation (Ma et al., 2022). By creating a nanocarrier with gold nanoparticles, antagomir-155, and a nuclear protein-specific aptamer, Kardani et al. (2020) were able to block miR-155. They found that TP53INP1 mRNA, a direct target protein of miR-155, increased in levels while miR-155 mRNA levels dramatically decreased. The utilization of exosomes is another interesting delivery method for miRNA. Because exosomes can effectively cross biological vectors and retain communication with target cells, using them as delivery vectors for miRNAs may be a viable way to overcome miRNA degradation *in vivo*. Exosomes can enhance the production of particular endogenous miRNAs and facilitate the regulation of several physiological processes, including death in cancer cells, according to their synthesis and targeting mechanisms (Fang et al., 2022). After being loaded with microRNA molecules in the exosome carriers, it was discovered that the resulting miRNA-126 loaded 231-Exo (miRNA-231-Exo) significantly inhibited the migration and proliferation of A549 lung cancer cells by blocking the PTEN/PI3K/AKT signaling pathway (Nie et al., 2020). Furthermore, animals that received intravenous treatment of miRNA-126-loaded exosomes experienced a strong lung homing effect.

##### 4.2.4.4 Potential therapies for other non-coding RNAs in breast cancer

CircRNAs control endocrine resistance by acting as miRNA sponges (Yi et al., 2023). In BC tissues, Xia et al. (2023) discovered that G3BP2 was overexpressed and miR-217 expression was decreased. The luciferase experiment confirmed that G3BP2 is a direct target of miR-217. BC cell movement is inhibited by G3BP2 expression inhibition. Through the circBACH1/miR-217/G3BP2 axis, paclitaxel-induced exosome circBACH1 controls BC cell stemness and migration by sponging miR-217 to increase G3BP expression. This offers a new therapeutic target for paclitaxel resistance and BC progression. In many instances, MiR-204-5p is downregulated in MCF-7 cells and BC patients (Liang et al., 2019). Jiang et al. (2023) showed that circRHOT1 serves as a sponge for miR-204-5p and stimulates the epithelial-mesenchymal transition (EMT) and invasion of breast cancer cells. Since miR-204-5p targets the protein arginine methyl transferase 5 (PRMT5) and exhibits the opposite expression pattern, they were able to reverse EMT by overexpressing PRMT5, which in turn reversed the effects of circRHOT1 knockdown on the expression of E-calcineurin, N-calcineurin, and poikilodulin, as well as on cell growth, apoptosis, wound healing and cell invasion. One RNA interference technique that can inhibit target genes is siRNA. Li et al. (2020) constructed an endosomal pH-responsive nanoparticle that contained cisplatin and Rac1 siRNA. This led to the effective delivery of cisplatin and Rac1 targeting oligonucleotides in breast cancers and displayed encouraging synergistic anticancer effects. Lipid-coated calcium phosphate nanoparticles were employed by Wu et al. (Wu Y. et al., 2019) to inhibit PD-1 and PD-L1. As a result, the siRNA can effectively enter the MCF-7 BC cell line and block the PD 1 ligand and receptor. A novel and practical genome editing technique, the clustered regulated interspaced short palindromic repeats (CRISPR)/Cas9 system is becoming a potent instrument for precision medicine (Behrouzian Fard et al., 2024). In contrast to EZH2 knockdown, which prevented MDA-MB-231 cells from proliferating and migrating *in vitro*, Mao et al. (Mao et al., 2023) used the CRISPR/Cas9 system to target EZH2 and suppress EZH2 mRNA and protein expression in MDA-MB-231 cells. Many experts believed that certain nanoparticles may be made for effective targeted distribution of CRISPR/Cas9 plasmids based on the function of CRISPR/Cas9 (Moitra et al., 2024).

#### 4.2.5 Combination therapy

Past research has explored the effectiveness of DNMT inhibitors and HDAC inhibitors in treating breast cancer. Nevertheless, these studies have demonstrated limited effectiveness even at the highest tolerated doses. As a result, epidrugs have been utilized alongside cytotoxic drugs, radiation treatment, targeted therapies, and hormonal treatments for breast cancer (Exman et al., 2019). Despite the potential, clinical trial outcomes have been disappointing due to systemic toxicity and limited effectiveness. Thus, identifying suitable epigenetic biomarkers is essential for personalized strategies and the targeted administration of epidrugs. Notably, HDAC inhibitors demonstrated enhanced antiproliferative effects in endocrine therapy for ER+ cells. The combination of azacytidine and entinostat, as well as HDAC inhibitor therapy on its own, resulted in the re-expression of ER and effective resistance to anti-estrogen treatments in ER-positive breast cancer (Buocikova et al., 2020). Furthermore, the BET inhibitor JQ1, either on its own or when paired with specific molecules that promote the downregulation of estrogen receptors, inhibited the proliferation of tamoxifen-resistant cells (Yellapu et al., 2022). Moreover, a synergistic effect from combination therapy has been shown in triple-negative breast cancer (TNBC). For example, a clinical study has explored the combined effectiveness of HDAC inhibitors and anti-HER2 treatment with trastuzumab (Laengle et al., 2020). Therefore, a suitable mix of medications can address oncogenic processes.

The oncogenic E2-ERα axis is the main target of endocrine treatments. In 1896, when BC patients’ tumors shrank after both ovaries had been surgically removed, steroid hormone signaling was first linked to the advancement of BC. This finding supported the use of endocrine therapy (Beatson, 1896). Endocrine therapy is considered to be the standard of care for ER^+^ BC, which includes three main types of treatments: aromatase inhibitors (AIs), selective estrogen receptor modulators (SERMs), and selective estrogen receptor degraders (SERDs). Endocrine therapy includes both strategies that directly target ERα and those that suppress estrogen production. Furthermore, next-generation ERα targeting treatments for ER+/HER2-metastasized BC are currently undergoing clinical trials as either single agents or in combination with other medications (Hanker et al., 2020).

For more than 30 years, tamoxifen has been remained the main treatment option for patients with both early and metastatic BC. It was the first ERα-targeted medication to receive clinical approval. Tamoxifen is a SERM that inhibits coactivator recruitment mediated by the LBD of ERα and competes with E2 for ERα binding. Through a ligand-independent mechanism, *in vitro*, it can also activate the AF1 domain, leading to slight transcriptional activation in absence of E2 and an incomplete block in presence of E2 stimulation (Liu et al., 2001). Through post-transcriptional modifications like CDK7, MAPK and mTOR50 phosphorylating serine 118 (pS118) in the AF1 domain, these agonistic effects are linked to ERα activation. Even though tamoxifen therapy is successful, one-third of women who receive 5 years of tamoxifen will experience recurrent disease within 15 years (Group, 2005). However, because most of these patients still express ERα, they are still susceptible to SERDs such as fulvestrant, which disrupts ERα dimerization and nuclear localization, leading to its degradation and a complete inhibition of ERα mediated transcriptional activity. The inhibition of transcription and subsequent degradation of ERα (Guan et al., 2019) are linked to fulvestrant-mediated ERα immobilization in the nuclear matrix. Patients with luminal BC who had not previously had hormone therapy participated in a phase III trial, which showed that fulvestrant treatment produces a better progression-free survival than AIs (Robertson et al., 2016). However, its therapeutic potential is limited by its weak physicochemical characteristics and the requirement for muscle administration (Guan et al., 2019). Clinical research is currently underway for a new class of ERα-targeting medicines that combine SERM and SERD characteristics, as well as new oral SERDs (Fanning and Greene, 2019). E2 is no longer produced in the ovaries of postmenopausal women. However, it is generated by the aromatization of testosterone and androstenedione in a number of tissues, such as the liver, subcutaneous fat, the normal breast cell stroma, and the fibroblasts and breast epithelial cells of primary BC. AIs can be categorized as either steroidal or non-steroidal and work by inhibiting aromatase activity to lower increased E2 levels in BC tissue. Non-steroidal AIs bind to aromatase both competitively and reversibly, whereas steroidal AIs bind irreversibly. Currently, two reversible non-steroidal AIs (letrozole, anastrozole) and one irreversible steroidal AI (exemestane) got their approval for clinical usage (Burstein, 2020). In contrast to tamoxifen, which is usually administered for patients with premenopausal BC, fulvestrant and AIs are primarily used for post-menopausal instances, either alone or in combination with other endocrine or targeted medicines such as CDK4/6 inhibitors, Other genetic changes, such as cyclin D1 overexpression in 50% of BC and CDKN2A loss, contribute to the course of the disease and the response to treatment, even though ERα is the main oncogenic driver in ER^+^ BC. For example, overexpression of cyclin D1 causes RB to become phosphorylated and CDK4/6 to become more activated, which in turn causes the cell cycle to advance through G1/S. The approval of targeted medications against PI3K (alpelisib), mTOR (everolimus), and CDK4/6 (palbociclib, riboci clib, and abemaciclib) after decades of endocrine monotherapy resulted in notable advancements in disease management. The effectiveness of CDK4/6 inhibition was shown in numerous clinical trials (Burstein, 2020). As a result, CDK4/6 inhibitors, either by themselves or in conjunction with AIs (letrozole) or fulvestrant, are now considered standard-of-care options for ER^+^/HER2-metastasized BC that are endocrine-sensitive or endocrine-resistant. Mechanisms of resistance to endocrine therapy and possible substitute methods. Even though endocrine therapy is effective in treating ER^+^ BC, resistance develops in nearly all patients who acquire metastases and in approximately 25% of patients with early-stage illness, which results in a poor clinical outcome (Jeselsohn et al., 2015). Resistance to endocrine therapy can be classified as either acquired or inherent (*de novo*). Patients with advanced breast cancer usually show clonally distinct progression at several places, which is caused by the selection of genetic changes under treatment pressure (Razavi et al., 2018). Clones with mutations in the drug target itself, mitogenic signaling pathways, and genes encoding epigenetic factors proliferate as a result of this selection pressure. Furthermore, as epigenetic enzymes are both oxygen and nutrition sensors, micro-environmental factors like hypoxia may change the epigenetic landscape and aid in the convergent evolution of the disease. In particular, clones with epigenetic machinery mutations show abnormalities in transcription, DNA repair, and replication. These errors result in malignant self-renewal, differentiation blockage, and cell death evasion, all of which increase tissue invasiveness. In the field of ER^+^ BC therapy, overcoming these results is a significant issue.

Given, endocrine therapy has established itself as a vital treatment option for breast tumors that respond to hormones. However, there is an urgent need to create techniques to tackle the apparently unavoidable resistant phenotype. The emerging new era of epigenetic-based therapeutics to screen and treat a variety of diseases, including BC, is evidenced by recent advancements in the epidrug field.

#### 4.2.6 Epi-drugs with nanotechnology

The instability, toxicity, and unintended effects of epidrugs are significant barriers to their effectiveness in treating solid tumors. Nanotechnology offers a means to specifically and directly target therapies at cancer cells. This approach allows for safer and more efficient delivery of epidrugs. Additionally, cancer nanotechnology can mitigate systemic toxicity by enhancing pharmacokinetics and selectively delivering anticancer medications to tumors. For example, nanoparticles such as albumin, membrane-camouflaged, lactoferrin and exosome-disguised nanoparticles represent innovative nanotechnologies aimed at directing tumor cells and modifying microenvironment of tumour in BC. Nano-delivery based on epigenetics promotes apoptosis and disrupts migration and proliferation (Zhang et al., 2023). Nanomedicine has the potential to enhance the effectiveness of tumor therapies that are resistant to treatment by integrating with next-generation epidrugs (Roberti et al., 2019). This indicates that utilizing nanotechnology in the medical field presents fresh possibilities to optimize drug delivery during epidemics, boost stability and solubility, and reduce off-target effects (Table 3).

**TABLE 3:** Epigenetic agents and their mechanism.

Epidrugs	Mechanism
Iadademstat	anti-SOX-2
Decitabine	demethylates DNA
Azacitidine	Hinders metastasis of BC to the brain and reduces the Wnt signaling pathway
Vorinostat	increase in the expression of PD-L1 mRNA
Guadecitabine	enhances the expression of PD-L1 and MHC class I while reducing the proliferation of tumor cells.
Liraglutide	decreased cell viability, migration, and DNMT activity
Simvastatin and Vorinostat	blocking Rab7 prenylation in xenograft mice *in vivo*
Letrozole	an aromatase inhibitor
Trichostatin A	strong inhibitor of pandeacetylase
Panobinostat	an HDAC inhibitor
Varprobic acid	Suppresses proliferation of HER2+ BC cells by increasing the expression of p21 WAF1
Azacytidine and Entinostat	Cause re-expression of ER and effective resistance to anti-estrogen treatments in ER-positive BC
BET inhibitor JQ1	either on its own or when paired with specific molecules that promote the downregulation of estrogen receptors, inhibited the proliferation of tamoxifen-resistant cells

### 4.3 Clinical trials

Connecting epigenetic research with practical medical use presents multiple obstacles. Converting discoveries from the lab into successful treatments demands not only a thorough knowledge of epigenetic processes but also comprehensive clinical trials to evaluate their effectiveness and safety. For instance, although epigenetic medications might demonstrate potential in cell cultures or animal experiments, their safety and effectiveness in human patients can be established solely through thorough clinical trials (Prabhu et al., 2024).

Preclinical research has shown that epi-drug DNMT inhibitors can diminish the tumorigeniNON-c potential of cancer stem cells by downregulating genes associated with stemness and differentiation. HDAC inhibitors reduce the functionality of cancer stem cells by affecting various critical genes that play a role in the maintenance of these cells, including those that code for β, γ-catenin, Stat3, and Notch1, which leads to a decrease in tumor formation (Xu et al., 2022). Clinical responses are generally non-cytotoxic and have been seen in individuals treated with low doses of DNMTi- and PD-1-conjugated therapeutic agents. These inhibitors have the potential to return the tumor microenvironment to a normal condition in colorectal cancer patients. Conversely, HDAC inhibitors in clinical studies have proven to be effective only in blood cancers, while trials involving solid tumors have not demonstrated significant results (Hogg et al., 2020). A phase II clinical trial has been carried out to investigate the effects of CC-486, a hypomethylating agent (HMA), and durvalumab in breast cancer treatment. The results of phase II clinical trials have indicated only limited clinical effectiveness. Treatment with anti-PD-1, anti-CTLA4, or both in combination has demonstrated tumor shrinkage and increased survival rates in clinical trials involving HER2/neurogenetic BC models. VPA, an antiepileptic medication typically prescribed for progressive prostate and breast cancers, specifically inhibits class I HDACs and helps decrease tumor growth and metastasis *in vivo*. Another study utilizing a mouse model of malignant pleural mesothelioma (MPM) revealed that a combination of drugs, including decitabine 2 and VPA, induced an anti-tumor immune response by promoting the expression of cancer-testis antigen (CTA) (Leclercq et al., 2011). In clinical studies, VPA has been paired with decitabine to investigate the immunogenic potential of the novel HDAC inhibitor. Furthermore, the mRNA expression levels of PD-L1, CTA, and retinoic acid-inducible protein I (RIG-I) were detected in MPM cells (Tomaselli et al., 2020).

Hydralazine has the potential to promote demethylation and reactivate tumor suppressor genes when used as a treatment for hypertension. This may increase the effectiveness of both biological and chemical therapies. In phase I, a clinical trial was undertaken to establish the safety of the dosage alongside standard cytotoxic chemotherapy in breast cancer patients. The findings from phase I revealed that hydralazine was well tolerated and showed no adverse effects on chemotherapy at doses of 200 mg or lower. Zambrano et al. demonstrated that demethylation occurred in up to 52% of the promoter region in selected tumor suppressor genes within a safe dose range to minimize toxicity. Further research on the combination of hydralazine and conventional cytotoxic chemotherapy is necessary, as these appear to be promising approaches to enhance effectiveness in phase II (Wu Y. S. et al., 2019; Cai et al., 2011).

A recent study performed immunohistochemical profiling of over 20 histone biomarkers, which included histone modifications, modifiers, and oncohistone mutations, across two cohorts of breast cancer tissues, a discovery cohort and a validation cohort, as well as healthy controls and cell line models. To assess the impact of the G9a small-molecule inhibitor in various breast cancer models, transcriptomic and cell growth analyses were carried out. Notable histone biomarkers such as H3K9me2, H3K36me2, and H3K79me showed differential expression among the breast cancer subtypes. H3K9me2 was identified as an independent marker for differentiating triple-negative breast cancer (TNBC) from other, less aggressive breast cancer subtypes, with increased expression linked to higher tumor grades and stages. Inhibition of G9a led to reduced cell proliferation and alteration of epithelial-mesenchymal transition pathways, particularly exhibiting the most pronounced effects in basal-like TNBC. Disruption in the regulation of oncogenes and tumor suppressors, such as TP53 and SATB1, was found in TNBC. This research emphasizes the context-dependent functions of G9a in breast cancer, indicating its potential as a target for therapy. The results lay the groundwork for epigenetic therapies tailored to specific subtypes to enhance outcomes for aggressive breast cancer types (Huo et al., 2025).

## 5 Limitations and prospects

There are several difficulties linked to the primary strategies of epidrugs concerning cytotoxicity, tolerance, selectivity, and potency. When patients with early-stage ER+ breast cancer are treated solely with epidrugs, one-third experience treatment resistance, and there is also drug resistance observed in TNBC (Jones et al., 2016; de Lera and Ganesan, 2016). Three approaches can be employed to address the limited effectiveness of individual epidrug targets: multiple-medication therapy (MMT), multi-compound medication (MCM), and multi-target-directed ligand (MTDLs) strategies. Implementing such strategies is anticipated to result in high efficacy, encompassing enhanced therapeutic outcomes, minimized side effects, and lowered risk of drug resistance (Li et al., 2017). MMT enhances the accessibility of chromatin to DNA-damaging chemotherapy agents and improves the effectiveness of various drugs. For example, methylation of CpG islands leads to resistance to therapy; however, using the MMT approach, the combination of zebulin and decitabine facilitates demethylation (Webster et al., 2017). MMT has the potential to enhance the therapeutic impact at reduced doses by reducing drug resistance and side effects in comparison to individual medications, enabling the selection of diverse drug dosages for tailored treatment. Likewise, MCM entails the combination of two or more active components, each serving a distinct purpose, and this approach is applied through the use of “polyvalent pills (Benedetti et al., 2015).” Ultimately, the MTDLs strategy represents the creation of a single molecule capable of simultaneously engaging with multiple targets. MTDLs are composite molecules that combine the action of HDAC inhibitors with that of other medications to target and combat cancer (Doostmohammadi et al., 2024). An alternative approach that can address the limitations of a single drug is the multi-drug strategy, which tends to be more effective. The combination of HDACi, romidepsin, cisplatin, and nivolumab demonstrated substantial efficacy in treating refractory metastatic TNBC. However, multi-drug therapies do not always yield positive results. For instance, there was no notable difference in the overall response rate and progression-free survival when using atezolizumab alone, and adverse effects were noted with the combination therapy (Xu et al., 2022). As a result, innovative strategies for utilizing epidrugs alongside different therapies to boost their antitumor effects need to be established.

## 6 Conclusion

Epigenetic has emerged as a crucial field in breast cancer, providing novel insights into disease progression and treatment resistance. This review highlights the potential of epigenetic biomarkers in improving early detection and patient stratification, paving the way for precision oncology. Despite the promising advancements, challenges such as the stability and reproducibility of epigenetic signatures, as well as the clinical translation of epi-drugs, remain to be addressed. Future research should focus on large-scale clinical trials to validate epigenetic biomarker and optimize combination therapies for enhanced therapeutic outcomes. Additionally, the integration of nanotechnology in epigenetic drug delivery holds promise for overcoming drug resistance and minimizing adverse effects. As our understanding of BC epigenetics continues to evolve, leveraging these molecular insights could lead to more effective, personalized treatment strategies, ultimately improving patient prognosis and survival rates. Moreover, public health education programs and awareness campaigns including Breast Cancer Awareness Month, community screening drives, and risk reduction workshops have been implemented globally to improve early detection and inform women at risk, highlighting the importance of integrating epigenetic knowledge into community-level education and prevention strategies.

## Data Availability

The raw data supporting the conclusions of this article will be made available by the author, without undue reservation.

## References

[B1] Adams B. D. Cowee D. M. White B. A. (2009). The role of miR-206 in the epidermal growth factor (EGF) induced repression of estrogen receptor-alpha (ERalpha) signaling and a luminal phenotype in MCF-7 breast cancer cells. Mol. Endocrinol. 23 (8), 1215–1230. 10.1210/me.2009-0062 19423651 PMC2718747

[B2] Agostini M. Enzo M. V. Bedin C. Belardinelli V. Goldin E. Del Bianco P. (2011). Circulating cell-free DNA: a promising marker of regional lymphonode metastasis in breast cancer patients. Cancer Biomarkers 11 (2-3), 89–98. 10.3233/CBM-2012-0263 23011155 PMC13016216

[B3] Alba-Bernal A. Lavado-Valenzuela R. Domínguez-Recio M. E. Jiménez-Rodriguez B. Queipo-Ortuño M. I. Alba E. (2020). Challenges and achievements of liquid biopsy technologies employed in early breast cancer. EBioMedicine 62, 103100. 10.1016/j.ebiom.2020.103100 33161226 PMC7670097

[B4] Ansari J. Shackelford R. E. El-Osta H. (2016). Epigenetics in non-small cell lung cancer: from basics to therapeutics. Transl. Lung Cancer Res. 5 (2), 155–171. 10.21037/tlcr.2016.02.02 27186511 PMC4858572

[B5] Arabkari V. Barua D. Hossain M. M. Webber M. Smith T. Gupta A. (2023). miRNA-378 is downregulated by XBP1 and inhibits growth and migration of luminal breast cancer cells. Int. J. Mol. Sci. 25 (1), 186. 10.3390/ijms25010186 38203358 PMC10778669

[B6] Ayipo Y. O. Ajiboye A. T. Osunniran W. A. Jimoh A. A. Mordi M. N. (2022). Epigenetic oncogenesis, biomarkers and emerging chemotherapeutics for breast cancer. Biochimica Biophysica Acta (BBA)-Gene Regul. Mech. 1865 (7), 194873. 10.1016/j.bbagrm.2022.194873 36064110

[B7] Baratta M. G. Schinzel A. C. Zwang Y. Bandopadhayay P. Bowman-Colin C. Kutt J. (2015). An in-tumor genetic screen reveals that the BET bromodomain protein, BRD4, is a potential therapeutic target in ovarian carcinoma. Proc. Natl. Acad. Sci. 112 (1), 232–237. 10.1073/pnas.1422165112 25535366 PMC4291641

[B8] Baylin S. B. Jones P. A. (2016). Epigenetic determinants of cancer. Cold Spring Harb. Perspect. Biol. 8 (9), a019505. 10.1101/cshperspect.a019505 27194046 PMC5008069

[B9] Beatson G. T. (1896). On the treatment of inoperable cases of carcinoma of the mamma: suggestions for a new method of treatment, with illustrative cases. Trans. Medico-Chirurgical Soc. Edinb. 15, 153–179. 29584099 PMC5518378

[B10] Behrouzian Fard G. Ahmadi M. H. Gholamin M. Amirfakhrian R. Saberi Teimourian E. Karimi M. A. (2024). CRISPR‐Cas9 technology: as an efficient genome modification tool in the cancer diagnosis and treatment. Biotechnol. Bioeng. 121 (2), 472–488. 10.1002/bit.28603 37986642

[B11] Bell C. C. Fennell K. A. Chan Y. C. Rambow F. Yeung M. M. Vassiliadis D. (2019). Targeting enhancer switching overcomes non-genetic drug resistance in acute myeloid leukaemia. Nat. Commun. 10 (1), 2723. 10.1038/s41467-019-10652-9 31222014 PMC6586637

[B12] Benedetti R. Conte M. Iside C. Altucci L. (2015). Epigenetic-based therapy: from single-to multi-target approaches. Int. J. Biochem. & Cell Biol. 69, 121–131. 10.1016/j.biocel.2015.10.016 26494003

[B13] Berdasco M. Esteller M. (2019). Clinical epigenetics: seizing opportunities for translation. Nat. Rev. Genet. 20 (2), 109–127. 10.1038/s41576-018-0074-2 30479381

[B14] Boi M. Gaudio E. Bonetti P. Kwee I. Bernasconi E. Tarantelli C. (2015). The BET bromodomain inhibitor OTX015 affects pathogenetic pathways in preclinical B-cell tumor models and synergizes with targeted drugs. Clin. Cancer Res. 21 (7), 1628–1638. 10.1158/1078-0432.CCR-14-1561 25623213

[B15] Brzeziańska E. Dutkowska A. Antczak A. (2013). The significance of epigenetic alterations in lung carcinogenesis. Mol. Biol. Rep. 40 (1), 309–325. 10.1007/s11033-012-2063-4 23086271 PMC3518808

[B16] Buocikova V. Rios-Mondragon I. Pilalis E. Chatziioannou A. Miklikova S. Mego M. (2020). Epigenetics in breast cancer therapy—new strategies and future nanomedicine perspectives. Cancers 12 (12), 3622. 10.3390/cancers12123622 33287297 PMC7761669

[B17] Burstein H. J. (2020). Systemic therapy for estrogen receptor–positive, HER2-negative breast cancer. N. Engl. J. Med. 383 (26), 2557–2570. 10.1056/NEJMra1307118 33369357

[B18] Butler C. Sprowls S. Szalai G. Arsiwala T. Saralkar P. Straight B. (2020). Hypomethylating agent azacitidine is effective in treating brain metastasis triple-negative breast cancer through regulation of DNA methylation of keratin 18 gene. Transl. Oncol. 13 (6), 100775. 10.1016/j.tranon.2020.100775 32408199 PMC7225776

[B19] Cai F.-F. Kohler C. Zhang B. Wang M. H. Chen W. J. Zhong X. Y. (2011). Epigenetic therapy for breast cancer. Int. J. Mol. Sci. 12 (7), 4465–4487. 10.3390/ijms12074465 21845090 PMC3155363

[B20] Cappellacci L. Perinelli D. R. Maggi F. Grifantini M. Petrelli R. (2020). Recent progress in histone deacetylase inhibitors as anticancer agents. Curr. Med. Chem. 27 (15), 2449–2493. 10.2174/0929867325666181016163110 30332940

[B21] Chaudhari R. Nasra S. Meghani N. Kumar A. (2022). MiR-206 conjugated gold nanoparticle based targeted therapy in breast cancer cells. Sci. Rep. 12 (1), 4713. 10.1038/s41598-022-08185-1 35304514 PMC8933417

[B22] Chen B. Sang Y. Song X. Zhang D. Wang L. Zhao W. (2021). Exosomal miR-500a-5p derived from cancer-associated fibroblasts promotes breast cancer cell proliferation and metastasis through targeting USP28. Theranostics 11 (8), 3932–3947. 10.7150/thno.53412 33664871 PMC7914354

[B23] Chen X. Luo R. Zhang Y. Ye S. Zeng X. Liu J. (2022). Long noncoding RNA DIO3OS induces glycolytic-dominant metabolic reprogramming to promote aromatase inhibitor resistance in breast cancer. Nat. Commun. 13 (1), 7160. 10.1038/s41467-022-34702-x 36418319 PMC9684133

[B24] Chen X. Ding J. C. Hu G. S. Shu X. Y. Liu Y. Du J. (2023). Estrogen‐Induced LncRNA, LINC02568, promotes estrogen receptor‐positive breast cancer development and drug resistance through both in trans and in cis mechanisms. Adv. Sci. 10 (25), 2206663. 10.1002/advs.202206663 37404090 PMC10477896

[B25] Cheng Y. Wang M. Ma X. Mo F. Yang S. (2019). Targeting epigenetic regulators for cancer therapy: mechanisms and advances in clinical trials. Signal Transduct. Target. Ther. 4 (1), 62. 10.1038/s41392-019-0095-0 31871779 PMC6915746

[B26] Chequin A. Costa L. E. de Campos F. F. Moncada A. D. B. de Lima L. T. F. Sledz L. R. (2021). Antitumoral activity of liraglutide, a new DNMT inhibitor in breast cancer cells *in vitro* and *in vivo* . Chemico-Biological Interact. 349, 109641. 10.1016/j.cbi.2021.109641 34534549

[B27] Coudé M.-M. Braun T. Berrou J. Dupont M. Bertrand S. Masse A. (2015). BET inhibitor OTX015 targets BRD2 and BRD4 and decreases c-MYC in acute leukemia cells. Oncotarget 6 (19), 17698–17712. 10.18632/oncotarget.4131 25989842 PMC4627339

[B28] Cuyàs E. Gumuzio J. Verdura S. Brunet J. Bosch-Barrera J. Martin-Castillo B. (2020). The LSD1 inhibitor iadademstat (ORY-1001) targets SOX2-driven breast cancer stem cells: a potential epigenetic therapy in luminal-B and HER2-positive breast cancer subtypes. Aging (Albany NY) 12 (6), 4794–4814. 10.18632/aging.102887 32191225 PMC7138538

[B29] Dai X. Li T. Bai Z. Yang Y. Liu X. Zhan J. (2015). Breast cancer intrinsic subtype classification, clinical use and future trends. Am. J. cancer Res. 5 (10), 2929–2943. 26693050 PMC4656721

[B30] Dawson M. A. Kouzarides T. (2012). Cancer epigenetics: from mechanism to therapy. cell 150 (1), 12–27. 10.1016/j.cell.2012.06.013 22770212

[B31] Dawson M. A. Prinjha R. K. Dittmann A. Giotopoulos G. Bantscheff M. Chan W. I. (2011). Inhibition of BET recruitment to chromatin as an effective treatment for MLL-fusion leukaemia. Nature 478 (7370), 529–533. 10.1038/nature10509 21964340 PMC3679520

[B32] de Almeida B. P. Apolónio J. D. Binnie A. Castelo-Branco P. (2019). Roadmap of DNA methylation in breast cancer identifies novel prognostic biomarkers. BMC cancer 19, 219–12. 10.1186/s12885-019-5403-0 30866861 PMC6416975

[B33] de Lera A. R. Ganesan A. (2016). Epigenetic polypharmacology: from combination therapy to multitargeted drugs. Clin. Epigenetics 8 (1), 105. 10.1186/s13148-016-0271-9 27752293 PMC5062873

[B34] Deng G. Zeng F. He Y. Meng Y. Sun H. Su J. (2022). EEF2K silencing inhibits tumour progression through repressing SPP1 and synergises with BET inhibitors in melanoma. Clin. Transl. Med. 12 (2), e722. 10.1002/ctm2.722 35184394 PMC8858631

[B35] Donnarumma E. Fiore D. Nappa M. Roscigno G. Adamo A. Iaboni M. (2017). Cancer-associated fibroblasts release exosomal microRNAs that dictate an aggressive phenotype in breast cancer. Oncotarget 8 (12), 19592–19608. 10.18632/oncotarget.14752 28121625 PMC5386708

[B36] Doostmohammadi A. Jooya H. Ghorbanian K. Gohari S. Dadashpour M. (2024). Potentials and future perspectives of multi-target drugs in cancer treatment: the next generation anti-cancer agents. Cell Commun. Signal 22 (1), 228. 10.1186/s12964-024-01607-9 38622735 PMC11020265

[B37] Echeverria G. V. Ge Z. Seth S. Zhang X. Jeter-Jones S. Zhou X. (2019). Resistance to neoadjuvant chemotherapy in triple-negative breast cancer mediated by a reversible drug-tolerant state. Sci. Transl. Med. 11 (488), eaav0936. 10.1126/scitranslmed.aav0936 30996079 PMC6541393

[B38] Ediriweera M. K. Tennekoon K. H. Samarakoon S. R. (2019). Emerging role of histone deacetylase inhibitors as anti-breast-cancer agents. Drug Discov. Today 24 (3), 685–702. 10.1016/j.drudis.2019.02.003 30776482

[B39] Egger G. (2018). Epigenetic biomarkers in cancer. ESMO open 3 (5), e000416. 10.1136/esmoopen-2018-000416 30116593 PMC6088343

[B40] Erdmann A. Halby L. Fahy J. Arimondo P. B. (2015). Targeting DNA methylation with small molecules: what's next? J. Med. Chem. 58 (6), 2569–2583. 10.1021/jm500843d 25406944

[B41] Exman P. Barroso-Sousa R. Tolaney S. M. (2019). Evidence to date: talazoparib in the treatment of breast cancer. Onco Targets Ther. 12, 5177–5187. 10.2147/OTT.S184971 31303769 PMC6612288

[B42] Fang Y. Yang C. Yu Z. Li X. Mu Q. Liao G. (2021). Natural products as LSD1 inhibitors for cancer therapy. Acta Pharm. Sin. B 11 (3), 621–631. 10.1016/j.apsb.2020.06.007 32837872 PMC7305746

[B43] Fang Z. Zhang X. Huang H. Wu J. (2022). Exosome based miRNA delivery strategy for disease treatment. Chin. Chem. Lett. 33 (4), 1693–1704. 10.1016/j.cclet.2021.11.050

[B44] Fanning S. W. Greene G. L. (2019). Next-generation ERα inhibitors for endocrine-resistant ER+ breast cancer. *Next-Generation ER α Inhibitors Endocrine-Resistant ER+ Breast Cancer.* Endocrinol. 160 (4), 759–769. 10.1210/en.2018-01095 30753408

[B45] Fernandez-Garcia D. Hills A. Page K. Hastings R. K. Toghill B. Goddard K. S. (2019). Plasma cell-free DNA (cfDNA) as a predictive and prognostic marker in patients with metastatic breast cancer. Breast Cancer Res. 21, 149–13. 10.1186/s13058-019-1235-8 31856868 PMC6924016

[B46] Filippakopoulos P. Qi J. Picaud S. Shen Y. Smith W. B. Fedorov O. (2010). Selective inhibition of BET bromodomains. Nature 468 (7327), 1067–1073. 10.1038/nature09504 20871596 PMC3010259

[B47] Filippakopoulos P. Picaud S. Mangos M. Keates T. Lambert J. P. Barsyte-Lovejoy D. (2012). Histone recognition and large-scale structural analysis of the human bromodomain family. Cell 149 (1), 214–231. 10.1016/j.cell.2012.02.013 22464331 PMC3326523

[B48] Garrido-Cano I. Adam-Artigues A. Lameirinhas A. Blandez J. F. Candela-Noguera V. Lluch A. (2023). Delivery of miR-200c-3p using tumor-targeted mesoporous silica nanoparticles for breast cancer therapy. ACS Appl. Mater. & Interfaces 15 (32), 38323–38334. 10.1021/acsami.3c07541 37549382 PMC10436244

[B49] Gilmer J.-J. (2020). Comb. Ther. Guadecitabine Immune Checkp. Inhibitors a Murine Triple-Negative Breast Cancer Model.

[B50] Ginsburg O. Yip C. H. Brooks A. Cabanes A. Caleffi M. Dunstan Yataco J. A. (2020). Breast cancer early detection: a phased approach to implementation. Cancer 126, 2379–2393. 10.1002/cncr.32887 32348566 PMC7237065

[B51] Giordano C. Accattatis F. M. Gelsomino L. Del Console P. Győrffy B. Giuliano M. (2023). miRNAs in the box: potential diagnostic role for extracellular vesicle-packaged miRNA-27a and miRNA-128 in breast cancer. Int. J. Mol. Sci. 24 (21), 15695. 10.3390/ijms242115695 37958677 PMC10649351

[B52] Goyal A. Sahu R. K. Kumar M. Sharma S. Qayyum S. Kaur N. (2019). p16 promoter methylation, expression, and its association with estrogen receptor, progesterone receptor, and human epidermal growth factor receptor 2 subtype of breast carcinoma. J. cancer Res. Ther. 15 (5), 1147–1154. 10.4103/jcrt.JCRT_472_18 31603125

[B53] Group E. B. C. T. C. (2005). Effects of chemotherapy and hormonal therapy for early breast cancer on recurrence and 15-year survival: an overview of the randomised trials. Lancet 365 (9472), 1687–1717. 10.1016/S0140-6736(05)66544-0 15894097

[B54] Guan J. Zhou W. Hafner M. Blake R. A. Chalouni C. Chen I. P. (2019). Therapeutic ligands antagonize estrogen receptor function by impairing its mobility. Cell 178 (4), 949–963.e18. 10.1016/j.cell.2019.06.026 31353221

[B55] Guo P. Chen W. Li H. Li L. (2018). The histone acetylation modifications of breast cancer and their therapeutic implications. Pathology & Oncol. Res. 24, 807–813. 10.1007/s12253-018-0433-5 29948617

[B56] Hanker A. B. Sudhan D. R. Arteaga C. L. (2020). Overcoming endocrine resistance in breast cancer. Cancer cell 37 (4), 496–513. 10.1016/j.ccell.2020.03.009 32289273 PMC7169993

[B57] Hogg S. J. Beavis P. A. Dawson M. A. Johnstone R. W. (2020). Targeting the epigenetic regulation of antitumour immunity. Nat. Rev. Drug Discov. 19 (11), 776–800. 10.1038/s41573-020-0077-5 32929243

[B58] Holliday R. (1994). Epigenetics: an overview. 10.1002/dvg.1020150602 7834903

[B59] Hu R. Li Y. Guo Y. Li X. Du S. Liao M. (2023). BRD4 inhibitor suppresses melanoma metastasis via the SPINK6/EGFR-EphA2 pathway. Pharmacol. Res. 187, 106609. 10.1016/j.phrs.2022.106609 36516883

[B60] Huang M. Zhang J. Yan C. Li X. Zhang J. Ling R. (2019). Small molecule HDAC inhibitors: promising agents for breast cancer treatment. Bioorg. Chem. 91, 103184. 10.1016/j.bioorg.2019.103184 31408831

[B61] Huo Z. Zhang S. Su G. Cai Y. Chen R. Jiang M. (2025). Immunohistochemical profiling of histone modification biomarkers identifies subtype-specific epigenetic signatures and potential drug targets in breast cancer. Int. J. Mol. Sci. 26 (2), 770. 10.3390/ijms26020770 39859484 PMC11765579

[B62] Ikeda K. Horie-Inoue K. Ueno T. Suzuki T. Sato W. Shigekawa T. (2015). miR-378a-3p modulates tamoxifen sensitivity in breast cancer MCF-7 cells through targeting GOLT1A. Sci. Rep. 5 (1), 13170. 10.1038/srep13170 26255816 PMC4530347

[B63] Jeselsohn R. Buchwalter G. De Angelis C. Brown M. Schiff R. (2015). ESR1 mutations—a mechanism for acquired endocrine resistance in breast cancer. Nat. Rev. Clin. Oncol. 12 (10), 573–583. 10.1038/nrclinonc.2015.117 26122181 PMC4911210

[B64] Jiang W. Yu Y. Ou J. Li Y. Zhu N. (2023). Exosomal circRNA RHOT1 promotes breast cancer progression by targeting miR-204-5p/PRMT5 axis. Cancer cell Int. 23 (1), 260. 10.1186/s12935-023-03111-5 37924099 PMC10623849

[B65] Jones P. A. Issa J.-P. J. Baylin S. (2016). Targeting the cancer epigenome for therapy. Nat. Rev. Genet. 17 (10), 630–641. 10.1038/nrg.2016.93 27629931

[B66] Kalia M. (2015). Biomarkers for personalized oncology: recent advances and future challenges. Metabolism 64 (3 Suppl. 1), S16–S21. 10.1016/j.metabol.2014.10.027 25468140

[B67] Kardani A. Yaghoobi H. Alibakhshi A. Khatami M. (2020). Inhibition of miR‐155 in MCF‐7 breast cancer cell line by gold nanoparticles functionalized with antagomir and AS1411 aptamer. J. Cell. Physiology 235 (10), 6887–6895. 10.1002/jcp.29584 32003016

[B68] Kaur M. Joshu C. E. Visvanathan K. Connor A. E. (2022). Trends in breast cancer incidence rates by race/ethnicity: patterns by stage, socioeconomic position, and geography in the United States, 1999‐2017. Cancer 128 (5), 1015–1023. 10.1002/cncr.34008 34731501 PMC9533488

[B69] Kim J.-H. Shin M. H. Kweon S. S. Park M. H. Yoon J. H. Lee J. S. (2010). Evaluation of promoter hypermethylation detection in serum as a diagnostic tool for breast carcinoma in Korean women. Gynecol. Oncol. 118 (2), 176–181. 10.1016/j.ygyno.2010.04.016 20466412

[B70] Kim A. Mo K. Kwon H. Choe S. Park M. Kwak W. (2023). Epigenetic regulation in breast cancer: insights on epidrugs. Epigenomes 7 (1), 6. 10.3390/epigenomes7010006 36810560 PMC9953240

[B71] Kopp F. Mendell J. T. (2018). Functional classification and experimental dissection of long noncoding RNAs. Cell 172 (3), 393–407. 10.1016/j.cell.2018.01.011 29373828 PMC5978744

[B72] Laengle J. Kabiljo J. Hunter L. Homola J. Prodinger S. Egger G. (2020). Histone deacetylase inhibitors valproic acid and vorinostat enhance trastuzumab-mediated antibody-dependent cell-mediated phagocytosis. J. Immunother. cancer 8 (1), e000195. 10.1136/jitc-2019-000195 31940587 PMC7057438

[B73] Laham-Karam N. Pinto G. P. Poso A. Kokkonen P. (2020). Transcription and translation inhibitors in cancer treatment. Front. Chem. 8, 276. 10.3389/fchem.2020.00276 32373584 PMC7186406

[B74] Langevin S. M. Kratzke R. A. Kelsey K. T. (2015). Epigenetics of lung cancer. Transl. Res. 165 (1), 74–90. 10.1016/j.trsl.2014.03.001 24686037 PMC4162853

[B75] Leclercq S. Gueugnon F. Boutin B. Guillot F. Blanquart C. Rogel A. (2011). A 5-aza-2'-deoxycytidine/valproate combination induces cytotoxic T-cell response against mesothelioma. Eur. Respir. J. 38 (5), 1105–1116. 10.1183/09031936.00081310 21540307

[B76] Levin A. A. (2019). Treating disease at the RNA level with oligonucleotides. N. Engl. J. Med. 380 (1), 57–70. 10.1056/NEJMra1705346 30601736

[B77] Li J. Hao D. Wang L. Wang H. Wang Y. Zhao Z. (2017). Epigenetic targeting drugs potentiate chemotherapeutic effects in solid tumor therapy. Sci. Rep. 7 (1), 4035. 10.1038/s41598-017-04406-0 28642588 PMC5481380

[B78] Li Q. Qin T. Bi Z. Hong H. Ding L. Chen J. (2020). Rac1 activates non-oxidative pentose phosphate pathway to induce chemoresistance of breast cancer. Nat. Commun. 11 (1), 1456. 10.1038/s41467-020-15308-7 32193458 PMC7081201

[B79] Liang A.-L. Zhang T. T. Zhou N. Wu C. Y. Lin M. H. Liu Y. J. (2016). MiRNA-10b sponge: an anti-breast cancer study *in vitro* . Oncol. Rep. 35 (4), 1950–1958. 10.3892/or.2016.4596 26820121 PMC4774667

[B80] Liang W. H. Yuan Z. Q. Qian X. L. Wang Z. H. (2019). DSCAM‐AS1 promotes tumor growth of breast cancer by reducing miR‐204‐5p and up‐regulating RRM2. Mol. Carcinog. 58 (4), 461–473. 10.1002/mc.22941 30457164

[B81] Liu H. Lee E. S. Deb Los Reyes A. Zapf J. W. Jordan V. C. (2001). Silencing and reactivation of the selective estrogen receptor modulator-estrogen receptor α complex. Cancer Res. 61 (9), 3632–3639. 11325832

[B82] Liu L. Sun L. Li C. Li X. Zhang Y. Yu Y. (2015). Quantitative detection of methylation of FHIT and BRCA1 promoters in the serum of ductal breast cancer patients. Bio-medical Mater. Eng. 26 (s1), S2217–S2222. 10.3233/BME-151527 26406001

[B83] Liu C. Lv D. Zhang X. Sun G. Bai Y. (2017). Hypermethylation of miRNA-589 promoter leads to upregulation of HDAC5 which promotes malignancy in non-small cell lung cancer. Int. J. Oncol. 50 (6), 2079–2090. 10.3892/ijo.2017.3967 28440397

[B84] Liu Z. Liu J. Ebrahimi B. Pratap U. P. He Y. Altwegg K. A. (2022). SETDB1 interactions with PELP1 contributes to breast cancer endocrine therapy resistance. Breast Cancer Res. 24 (1), 26. 10.1186/s13058-022-01520-4 35395812 PMC8991965

[B85] Liu X X. Zhang G. Yu T. He J. Liu J. Chai X. (2022). Exosomes deliver lncRNA DARS-AS1 siRNA to inhibit chronic unpredictable mild stress-induced TNBC metastasis. Cancer Lett. 543, 215781. 10.1016/j.canlet.2022.215781 35688263

[B86] Liu X. Zhang G. Yu T. Liu J. Chai X. Yin D. (2023). CL4-modified exosomes deliver lncRNA DARS-AS1 siRNA to suppress triple-negative breast cancer progression and attenuate doxorubicin resistance by inhibiting autophagy. Int. J. Biol. Macromol. 250, 126147. 10.1016/j.ijbiomac.2023.126147 37544559

[B87] Louie A. D. Huntington K. Carlsen L. Zhou L. El-Deiry W. S. (2021). Integrating molecular biomarker inputs into development and use of clinical cancer therapeutics. Front. Pharmacol. 12, 747194. 10.3389/fphar.2021.747194 34737704 PMC8560682

[B88] Luo F. Zhang M. Sun B. Xu C. Yang Y. Zhang Y. (2024). LINC00115 promotes chemoresistant breast cancer stem-like cell stemness and metastasis through SETDB1/PLK3/HIF1α signaling. Mol. Cancer 23 (1), 60. 10.1186/s12943-024-01975-3 38520019 PMC10958889

[B89] Ma C. He D. Tian P. Wang Y. He Y. Wu Q. (2022). miR-182 targeting reprograms tumor-associated macrophages and limits breast cancer progression. Proc. Natl. Acad. Sci. 119 (6), e2114006119. 10.1073/pnas.2114006119 35105806 PMC8833194

[B90] Mahendran G. Shangaradas A. D. Romero-Moreno R. Wickramarachchige Dona N. Sarasija S. H. G. S. Perera S. (2024). Unlocking the epigenetic code: new insights into triple-negative breast cancer. Front. Oncol. 14, 1499950. 10.3389/fonc.2024.1499950 39744000 PMC11688480

[B91] Mao Q. Wu P. Li H. Fu X. Gao X. Yang L. (2023). CRISPR/Cas9-mediated EZH2 knockout suppresses the proliferation and migration of triple-negative breast cancer cells. Oncol. Lett. 26 (2), 343. 10.3892/ol.2023.13929 37427349 PMC10326815

[B92] Martínez-Galán J. (2014). ESR1 gene promoter region methylation in free circulating DNA and its correlation with estrogen receptor protein expression in tumor tissue in breast cancer patients. BMC cancer 14, 1–8. 24495356 10.1186/1471-2407-14-59 PMC3922625

[B93] Mastoraki S. Strati A. Tzanikou E. Chimonidou M. Politaki E. Voutsina A. (2018). ESR1 methylation: a liquid biopsy–based epigenetic assay for the follow-up of patients with metastatic breast cancer receiving endocrine treatment. Clin. Cancer Res. 24 (6), 1500–1510. 10.1158/1078-0432.CCR-17-1181 29284708

[B94] Melo S. A. Sugimoto H. O'Connell J. T. Kato N. Villanueva A. Vidal A. (2014). Cancer exosomes perform cell-independent microRNA biogenesis and promote tumorigenesis. Cancer cell 26 (5), 707–721. 10.1016/j.ccell.2014.09.005 25446899 PMC4254633

[B95] Merrill N. M. Lachacz E. J. Vandecan N. M. Ulintz P. J. Bao L. Lloyd J. P. (2020). Molecular determinants of drug response in TNBC cell lines. Breast cancer Res. Treat. 179 (2), 337–347. 10.1007/s10549-019-05473-9 31655920 PMC7323911

[B96] Moitra P. Skrodzki D. Molinaro M. Gunaseelan N. Sar D. Aditya T. (2024). Context-responsive nanoparticle derived from synthetic zwitterionic ionizable phospholipids in targeted CRISPR/Cas9 therapy for basal-like breast cancer. ACS nano 18 (12), 9199–9220. 10.1021/acsnano.4c01400 38466962

[B97] Mollaei H. Safaralizadeh R. Rostami Z. (2019). MicroRNA replacement therapy in cancer. J. Cell. physiology 234 (8), 12369–12384. 10.1002/jcp.28058 30605237 PMC13484118

[B98] Nandy D. Rajam S. M. Dutta D. (2020). A three layered histone epigenetics in breast cancer metastasis. Cell & Biosci. 10 (1), 52. 10.1186/s13578-020-00415-1 32257110 PMC7106732

[B99] Nemeth K. Bayraktar R. Ferracin M. Calin G. A. (2024). Non-coding RNAs in disease: from mechanisms to therapeutics. Nat. Rev. Genet. 25 (3), 211–232. 10.1038/s41576-023-00662-1 37968332

[B100] Nie H. Xie X. Zhang D. Zhou Y. Li B. Li F. (2020). Use of lung-specific exosomes for miRNA-126 delivery in non-small cell lung cancer. Nanoscale 12 (2), 877–887. 10.1039/c9nr09011h 31833519

[B101] Nounou M. I. ElAmrawy F. Ahmed N. Abdelraouf K. Goda S. Syed-Sha-Qhattal H. (2015). Breast cancer: conventional diagnosis and treatment modalities and recent patents and technologies. Breast cancer basic Clin. Res. 9, 17–34. 10.4137/BCBCR.S29420 26462242 PMC4589089

[B102] Nwosu I. O. Piccolo S. R. (2024). A comprehensive meta-analysis of breast cancer gene expression. bioRxiv. 2024.08. 30.610515.

[B103] Page M. J. McKenzie J. E. Bossuyt P. M. Boutron I. Hoffmann T. C. Mulrow C. D. (2021). The PRISMA 2020 statement: an updated guideline for reporting systematic reviews. BMJ 372, n71. 10.1136/bmj.n71 33782057 PMC8005924

[B104] Panagopoulou M. Karaglani M. Balgkouranidou I. Biziota E. Koukaki T. Karamitrousis E. (2019). Circulating cell-free DNA in breast cancer: size profiling, levels, and methylation patterns lead to prognostic and predictive classifiers. Oncogene 38 (18), 3387–3401. 10.1038/s41388-018-0660-y 30643192

[B105] Parrella P. (2018). The value of epigenetic biomarkers in breast cancer. Taylor & Francis, 937–940. 10.2217/bmm-2018-0187 30041537

[B106] Pasculli B. Barbano R. Parrella P. (2018). “Epigenetics of breast cancer: biology and clinical implication in the era of precision medicine,” in Seminars in cancer biology (Elsevier). 10.1016/j.semcancer.2018.01.007 29339244

[B107] Patra S. Praharaj P. P. Klionsky D. J. Bhutia S. K. (2022). Vorinostat in autophagic cell death: a critical insight into autophagy-mediated,-associated and-dependent cell death for cancer prevention. Drug Discov. today 27 (1), 269–279. 10.1016/j.drudis.2021.08.004 34400351 PMC8714665

[B108] Peled M. Agassi R. Czeiger D. Ariad S. Riff R. Rosenthal M. (2020). Cell-free DNA concentration in patients with clinical or mammographic suspicion of breast cancer. Sci. Rep. 10 (1), 14601. 10.1038/s41598-020-71357-4 32884019 PMC7471679

[B109] Penna I. Gigoni A. Costa D. Vella S. Russo D. Poggi A. (2016). The inhibition of 45A ncRNA expression reduces tumor formation, affecting tumor nodules compactness and metastatic potential in neuroblastoma cells. Oncotarget 8 (5), 8189–8205. 10.18632/oncotarget.14138 28029658 PMC5352393

[B110] Piha-Paul S. A. Hann C. L. French C. A. Cousin S. Braña I. Cassier P. A. (2020). Phase 1 study of molibresib (GSK525762), a bromodomain and extra-terminal domain protein inhibitor, in NUT carcinoma and other solid tumors. JNCI Cancer Spectr. 4 (2), pkz093. 10.1093/jncics/pkz093 32328561 PMC7165800

[B111] Prabhu K. S. Sadida H. Q. Kuttikrishnan S. Junejo K. Bhat A. A. Uddin S. (2024). Beyond genetics: exploring the role of epigenetic alterations in breast cancer. Pathology - Res. Pract. 254, 155174. 10.1016/j.prp.2024.155174 38306863

[B112] Qin G. Li Y. Xu X. Wang X. Zhang K. Tang Y. (2019). Panobinostat (LBH589) inhibits Wnt/β-catenin signaling pathway via upregulating APCL expression in breast cancer. Cell. Signal. 59, 62–75. 10.1016/j.cellsig.2019.03.014 30880222

[B113] Rao X. Qiao Z. Yang Y. Deng Y. Zhang Z. Yu X. (2024). Unveiling epigenetic vulnerabilities in triple-negative breast cancer through 3D organoid drug screening. Pharmaceuticals 17 (2), 225. 10.3390/ph17020225 38399440 PMC10892330

[B114] Razavi P. Chang M. T. Xu G. Bandlamudi C. Ross D. S. Vasan N. (2018). The genomic landscape of endocrine-resistant advanced breast cancers. Cancer cell 34 (3), 427–438.e6. 10.1016/j.ccell.2018.08.008 30205045 PMC6327853

[B115] Richart L. Margueron R. (2020). Drugging histone methyltransferases in cancer. Curr. Opin. Chem. Biol. 56, 51–62. 10.1016/j.cbpa.2019.11.009 31981999

[B116] Roberti A. Valdes A. F. Torrecillas R. Fraga M. F. Fernandez A. F. (2019). Epigenetics in cancer therapy and nanomedicine. Clin. Epigenetics 11 (1), 81. 10.1186/s13148-019-0675-4 31097014 PMC6524244

[B117] Robertson J. F. Bondarenko I. M. Trishkina E. Dvorkin M. Panasci L. Manikhas A. (2016). Fulvestrant 500 mg versus anastrozole 1 mg for hormone receptor-positive advanced breast cancer (FALCON): an international, randomised, double-blind, phase 3 trial. Lancet 388 (10063), 2997–3005. 10.1016/S0140-6736(16)32389-3 27908454

[B118] Rohanizadegan M. (2018). Analysis of circulating tumor DNA in breast cancer as a diagnostic and prognostic biomarker. Cancer Genet. 228, 159–168. 10.1016/j.cancergen.2018.02.002 29572011 PMC6108954

[B119] Salas L. A. Lundgren S. N. Browne E. P. Punska E. C. Anderton D. L. Karagas M. R. (2020). Prediagnostic breast milk DNA methylation alterations in women who develop breast cancer. Hum. Mol. Genet. 29 (4), 662–673. 10.1093/hmg/ddz301 31943067 PMC7068171

[B120] Salta S. P Nunes S. Fontes-Sousa M. Lopes P. Freitas M. Caldas M. (2018). A DNA methylation-based test for breast cancer detection in circulating cell-free DNA. J. Clin. Med. 7 (11), 420. 10.3390/jcm7110420 30405052 PMC6262630

[B121] Sarvari P. Ramírez-Díaz I. Mahjoubi F. Rubio K. (2022). Advances of epigenetic biomarkers and epigenome editing for early diagnosis in breast cancer. Int. J. Mol. Sci. 23 (17), 9521. 10.3390/ijms23179521 36076918 PMC9455804

[B122] Shan M. Yin H. Li J. Li X. Wang D. Su Y. (2016). Detection of aberrant methylation of a six-gene panel in serum DNA for diagnosis of breast cancer. Oncotarget 7 (14), 18485–18494. 10.18632/oncotarget.7608 26918343 PMC4951303

[B123] Sher G. Salman N. A. Khan A. Q. Prabhu K. S. Raza A. Kulinski M. (2022). Epigenetic and breast cancer therapy: promising diagnostic and therapeutic applications. Seminars Cancer Biol. 83, 152–165. 10.1016/j.semcancer.2020.08.009 32858230

[B124] Song L. Bretz A. C. Gravemeyer J. Spassova I. Muminova S. Gambichler T. (2021). The HDAC inhibitor domatinostat promotes cell-cycle arrest, induces apoptosis, and increases immunogenicity of merkel cell carcinoma cells. J. Investigative Dermatology 141 (4), 903–912.e4. 10.1016/j.jid.2020.08.023 33002502 PMC7987731

[B125] Stone A. Zotenko E. Locke W. J. Korbie D. Millar E. K. A. Pidsley R. (2015). DNA methylation of oestrogen-regulated enhancers defines endocrine sensitivity in breast cancer. Nat. Commun. 6 (1), 7758. 10.1038/ncomms8758 26169690 PMC4510968

[B126] Su Y. Hopfinger N. R. Nguyen T. D. Pogash T. J. Santucci-Pereira J. Russo J. (2018). Epigenetic reprogramming of epithelial mesenchymal transition in triple negative breast cancer cells with DNA methyltransferase and histone deacetylase inhibitors. J. Exp. & Clin. Cancer Res. 37, 314–318. 10.1186/s13046-018-0988-8 30547810 PMC6295063

[B127] Sulewska A. Pilz L. Manegold C. Ramlau R. Charkiewicz R. Niklinski J. (2023). A systematic review of progress toward unlocking the power of epigenetics in NSCLC: latest updates and perspectives. Cells 12 (6), 905. 10.3390/cells12060905 36980246 PMC10047383

[B128] Sun M. Liu X. H. Wang K. M. Nie F. q. Kong R. Yang J. s. (2014). Downregulation of BRAF activated non-coding RNA is associated with poor prognosis for non-small cell lung cancer and promotes metastasis by affecting epithelial-mesenchymal transition. Mol. cancer 13, 68–12. 10.1186/1476-4598-13-68 24655544 PMC3998010

[B129] Swellam M. Abdelmaksoud M. D. E. Sayed Mahmoud M. Ramadan A. Abdel-Moneem W. Hefny M. M. (2015). Aberrant methylation of APC and RARβ2 genes in breast cancer patients. Iubmb Life 67 (1), 61–68. 10.1002/iub.1346 25684670

[B130] Tang Q. Cheng J. Cao X. Surowy H. Burwinkel B. (2016). Blood-based DNA methylation as biomarker for breast cancer: a systematic review. Clin. epigenetics 8, 115–118. 10.1186/s13148-016-0282-6 27895805 PMC5109688

[B131] Terranova-Barberio M. Thomas S. Ali N. Pawlowska N. Park J. Krings G. (2017). HDAC inhibition potentiates immunotherapy in triple negative breast cancer. Oncotarget 8 (69), 114156–114172. 10.18632/oncotarget.23169 29371976 PMC5768393

[B132] Thomas M. L. Marcato P. (2018). Epigenetic modifications as biomarkers of tumor development, therapy response, and recurrence across the cancer care continuum. Cancers 10 (4), 101. 10.3390/cancers10040101 29614786 PMC5923356

[B133] Tomaselli D. Lucidi A. Rotili D. Mai A. (2020). Epigenetic polypharmacology: a new frontier for epi-drug discovery. Med. Res. Rev. 40 (1), 190–244. 10.1002/med.21600 31218726 PMC6917854

[B134] Unger‐Saldaña K. Miranda A. Zarco-Espinosa G. Mainero-Ratchelous F. Bargalló-Rocha E. Miguel Lázaro-León J. (2015). Health system delay and its effect on clinical stage of breast cancer: multicenter study. Cancer 121 (13), 2198–2206. 10.1002/cncr.29331 25809536 PMC6681165

[B135] Vernier M. McGuirk S. Dufour C. R. Wan L. Audet-Walsh E. St-Pierre J. (2020). Inhibition of DNMT1 and ERRα crosstalk suppresses breast cancer via derepression of IRF4. Oncogene 39 (41), 6406–6420. 10.1038/s41388-020-01438-1 32855526 PMC7544553

[B136] Vieira A. F. Schmitt F. (2018). An update on breast cancer multigene prognostic tests—emergent clinical biomarkers. Front. Med. 5, 248. 10.3389/fmed.2018.00248 30234119 PMC6131478

[B137] Wang L. (2017). Early diagnosis of breast cancer. Sensors 17 (7), 1572. 10.3390/s17071572 28678153 PMC5539491

[B138] Wang X. Liu Z. Zhang L. Yang Z. Chen X. Luo J. (2018). Targeting deubiquitinase USP28 for cancer therapy. Cell death & Dis. 9 (2), 186. 10.1038/s41419-017-0208-z 29415985 PMC5833459

[B139] Wang W.-T. Han C. Sun Y. M. Chen T. Q. Chen Y. Q. (2019). Noncoding RNAs in cancer therapy resistance and targeted drug development. J. Hematol. & Oncol. 12 (1), 55. 10.1186/s13045-019-0748-z 31174564 PMC6556047

[B140] Wang X. Chen S. Shen T. Lu H. Xiao D. Zhao M. (2020). Trichostatin A reverses epithelial-mesenchymal transition and attenuates invasion and migration in MCF-7 breast cancer cells. Exp. Ther. Med. 19 (3), 1687–1694. 10.3892/etm.2020.8422 32104221 PMC7027139

[B141] Wang Y. Pan X. Li Y. Wang R. Yang Y. Jiang B. (2021). CUL4B renders breast cancer cells tamoxifen‐resistant via miR‐32‐5p/ER‐α36 axis. J. Pathology 254 (2), 185–198. 10.1002/path.5657 33638154

[B142] Wawruszak A. Halasa M. Okon E. Kukula-Koch W. Stepulak A. (2021). Valproic acid and breast cancer: state of the art in 2021. Cancers (Basel) 13 (14), 3409. 10.3390/cancers13143409 34298623 PMC8306563

[B143] Webster R. Castellano J. M. Onuma O. K. (2017). Putting polypills into practice: challenges and lessons learned. Lancet 389 (10073), 1066–1074. 10.1016/S0140-6736(17)30558-5 28290996

[B144] Winkle M. El-Daly S. M. Fabbri M. Calin G. A. (2021). Noncoding RNA therapeutics—challenges and potential solutions. Nat. Rev. Drug Discov. 20 (8), 629–651. 10.1038/s41573-021-00219-z 34145432 PMC8212082

[B145] Wong K. K. (2021). “DNMT1: a key drug target in triple-negative breast cancer,” in Seminars in cancer biology (Elsevier). 10.1016/j.semcancer.2020.05.010 32461152

[B146] Wortzel I. Dror S. Kenific C. M. Lyden D. (2019). Exosome-mediated metastasis: communication from a distance. Dev. cell 49 (3), 347–360. 10.1016/j.devcel.2019.04.011 31063754

[B147] Wu Y. Gu W. Li J. Chen C. Xu Z. P. (2019). Silencing PD-1 and PD-L1 with nanoparticle-delivered small interfering RNA increases cytotoxicity of tumor-infiltrating lymphocytes. Nanomedicine 14 (8), 955–967. 10.2217/nnm-2018-0237 30901292

[B148] Wu Y. S. Lee Z. Y. Chuah L. H. Mai C. W. Ngai S. C. (2019). Epigenetics in metastatic breast cancer: its regulation and implications in diagnosis, prognosis and therapeutics. Curr. Cancer Drug Targets 19 (2), 82–100. 10.2174/1568009618666180430130248 29714144

[B149] Wu C.-H. Tsai Y. C. Tsai T. H. Kuo K. L. Su Y. F. Chang C. H. (2021). Valproic acid reduces vasospasm through modulation of Akt phosphorylation and attenuates neuronal apoptosis in subarachnoid hemorrhage rats. Int. J. Mol. Sci. 22 (11), 5975. 10.3390/ijms22115975 34205883 PMC8198375

[B150] Xia W. Chen W. Ni C. Meng X. Wu J. Yang Q. (2023). Chemotherapy-induced exosomal circBACH1 promotes breast cancer resistance and stemness via miR-217/G3BP2 signaling pathway. Breast Cancer Res. 25 (1), 85. 10.1186/s13058-023-01672-x 37461019 PMC10351125

[B151] Xiong Q. Zhang Y. (2023). Small RNA modifications: regulatory molecules and potential applications. J. Hematol. & Oncol. 16 (1), 64. 10.1186/s13045-023-01466-w 37349851 PMC10286502

[B152] Xu Z. Sandler D. P. Taylor J. A. (2020). Blood DNA methylation and breast cancer: a prospective case-cohort analysis in the sister study. JNCI J. Natl. Cancer Inst. 112 (1), 87–94. 10.1093/jnci/djz065 30989176 PMC7489106

[B153] Xu Y. Li P. Liu Y. Xin D. Lei W. Liang A. (2022). Epi-immunotherapy for cancers: rationales of epi-drugs in combination with immunotherapy and advances in clinical trials. Cancer Commun. (Lond) 42 (6), 493–516. 10.1002/cac2.12313 35642676 PMC9198339

[B154] Yamashita R. Sato M. Kakumu T. Hase T. Yogo N. Maruyama E. (2015). Growth inhibitory effects of miR‐221 and miR‐222 in non‐small cell lung cancer cells. Cancer Med. 4 (4), 551–564. 10.1002/cam4.412 25641933 PMC4402070

[B155] Yang R. Pfütze K. Zucknick M. Sutter C. Wappenschmidt B. Marme F. (2015). DNA methylation array analyses identified breast cancer‐associated HYAL2 methylation in peripheral blood. Int. J. cancer 136 (8), 1845–1855. 10.1002/ijc.29205 25213452

[B156] Yang Q. Zhao S. Shi Z. Cao L. Liu J. Pan T. (2021). Chemotherapy-elicited exosomal miR-378a-3p and miR-378d promote breast cancer stemness and chemoresistance via the activation of EZH2/STAT3 signaling. J. Exp. & Clin. Cancer Res. 40 (1), 120. 10.1186/s13046-021-01901-1 33823894 PMC8022546

[B157] Yang L. Wang M. Wang Y. Zhu Y. Wang J. Wu M. (2024). LINC00460-FUS-MYC feedback loop drives breast cancer metastasis and doxorubicin resistance. Oncogene 43 (17), 1249–1262. 10.1038/s41388-024-02972-y 38418543

[B158] Yardim-Akaydin S. Karahalil B. Baytas S. N. (2022). New therapy strategies in the management of breast cancer. Drug Discov. Today 27 (6), 1755–1762. 10.1016/j.drudis.2022.03.014 35337961

[B159] Yellapu N. K. Ly T. Sardiu M. E. Pei D. Welch D. R. Thompson J. A. (2022). Synergistic anti-proliferative activity of JQ1 and GSK2801 in triple-negative breast cancer. BMC Cancer 22 (1), 627. 10.1186/s12885-022-09690-2 35672711 PMC9173973

[B160] Yi J. Wang L. Hu G. S. Zhang Y. Y. Du J. Ding J. C. (2023). CircPVT1 promotes ER‐positive breast tumorigenesis and drug resistance by targeting ESR1 and MAVS. EMBO J. 42 (10), e112408. 10.15252/embj.2022112408 37009655 PMC10183818

[B161] Yu J. Qin B. Moyer A. M. Nowsheen S. Liu T. Qin S. (2018). DNA methyltransferase expression in triple-negative breast cancer predicts sensitivity to decitabine. J. Clin. investigation 128 (6), 2376–2388. 10.1172/JCI97924 29708513 PMC5983332

[B162] Zhang G. Smith S. G. Zhou M.-M. (2015). Discovery of chemical inhibitors of human bromodomains. Chem. Rev. 115 (21), 11625–11668. 10.1021/acs.chemrev.5b00205 26492937

[B163] Zhang J. Li S. Li L. Li M. Guo C. Yao J. (2015). Exosome and exosomal microRNA: trafficking, sorting, and function. Genomics, proteomics & Bioinforma. 13 (1), 17–24. 10.1016/j.gpb.2015.02.001 25724326 PMC4411500

[B164] Zhang J. Huang L. Hu K. (2023). Emerging epigenetic-based nanotechnology for cancer therapy: modulating the tumor microenvironment. Adv. Sci. (Weinh) 10 (7), e2206169. 10.1002/advs.202206169 36599655 PMC9982594

[B165] Zhou X. Liu Z. Wang H. Liu X. Zhou Z. Tang J. (2019). SAHA (vorinostat) facilitates functional polymer-based gene transfection via upregulation of ROS and synergizes with TRAIL gene delivery for cancer therapy. J. drug Target. 27 (3), 306–314. 10.1080/1061186X.2018.1519028 30188217

[B166] Zubor P. Kubatka P. Kajo K. Dankova Z. Polacek H. Bielik T. (2019). Why the gold standard approach by mammography demands extension by multiomics? Application of liquid biopsy miRNA profiles to breast cancer disease management. Int. J. Mol. Sci. 20 (12), 2878. 10.3390/ijms20122878 31200461 PMC6627787

[B167] Zucchetti B. Shimada A. K. Katz A. Curigliano G. (2019). The role of histone deacetylase inhibitors in metastatic breast cancer. Breast 43, 130–134. 10.1016/j.breast.2018.12.001 30553187

